# The Human Placental Sexome Differs between Trophoblast Epithelium and Villous Vessel Endothelium

**DOI:** 10.1371/journal.pone.0079233

**Published:** 2013-10-29

**Authors:** Silvija Cvitic, Mark S. Longtine, Hubert Hackl, Karin Wagner, Michael D. Nelson, Gernot Desoye, Ursula Hiden

**Affiliations:** 1 Department of Obstetrics and Gynecology, Medical University of Graz, Graz, Austria; 2 Department of Obstetrics and Gynecology, Washington University, St. Louis, Missouri, United States of America; 3 Division of Bioinformatics, Biocenter, Innsbruck Medical University, Innsbruck, Austria; 4 Center for Medical Research, Medical University of Graz, Graz, Austria; Centro Cardiologico Monzino IRCCS, Italy

## Abstract

Molecular mechanisms underlying sexual dimorphism in mammals, fetal sex influences on intrauterine development, and the sex-biased susceptibility for selected diseases in adulthood are novel areas of current research. As importantly, two decades of multifaceted research has established that susceptibility to many adult disorders originates *in utero*, commonly secondary to the effects of placental dysfunction. We hypothesized that fetal sex influences gene expression and produces functional differences in human placentas. We thus extended previous studies on sexual dimorphism in mammals, which used RNA isolated from whole tissues, to investigate the effects of sex on four cell-phenotypes within a single key tissue, human placental villi. The cells studied included cytotrophoblasts, syncytiotrophoblast, arterial and venous endothelial cells. The cells were isolated from placentas of male or female fetuses and subjected to microarray analysis. We found that fetal sex differentially affected gene expression in a cell-phenotype dependent manner among all four cell-phenotypes. The markedly enriched pathways in males were identified to be signaling pathways for graft-versus-host disease as well as the immune and inflammatory systems that parallel the reported poorer outcome of male fetuses. Our study is the first to compare global gene expression by microarray analysis in purified, characterized, somatic cells from a single human tissue, i.e. placental villi. Importantly, our findings demonstrate that there are cell-phenotype specific, and tissue-specific, sex-biased responses in the human placenta, suggesting fetal sex should be considered as an independent variable in gene expression analysis of human placental villi.

## Introduction

Males and females express multiple phenotypic differences, but recent attention has focused on molecular mechanisms underlying sexual dimorphism in mammals and the sex-biased susceptibility for selected diseases in adulthood. Moreover, predisposition to many adult diseases originates *in utero*, secondary to placental dysfunction, a hypothesis commonly referred to as the Developmental Origins of Health and Disease (DOHaD). Importantly, fetal sex influences *in utero* development in both uncomplicated pregnancies and pregnancies with sub-optimal outcomes. For example, male fetuses have a higher risk for peri- and postnatal mortality and are generally larger than female [1]–[4]. In addition, male fetuses in pregnancy maladies have poorer outcomes than female fetuses [5], [6] and are more likely to develop hypertension, diabetes mellitus, or metabolic syndrome [7], [8].

**Figure 1:**
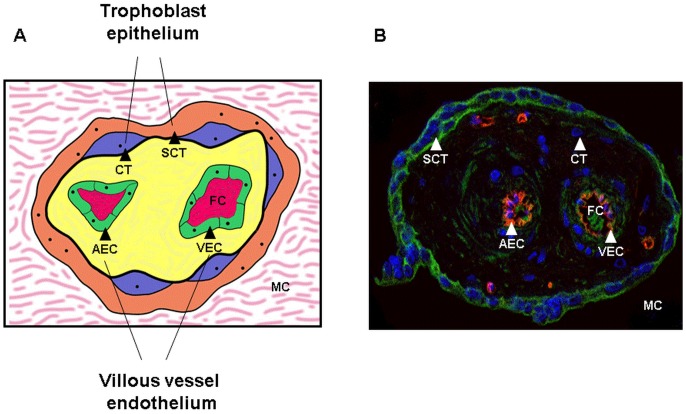
Placental villi. Schematic representation (A) and an immunohistochemical staining (B) of a villous cross section showing two main placental compartments. The trophoblast epithelium i.e., syncytiotrophoblast (SCT) and cytotrophoblasts (CT) (syncytial progenitors) is exposed to the maternal circulation (MC) while the fetal circulation (FC) is in direct contact with villous vessel endothelium, composed of arterial (AEC) and venous endothelial cells (AEC).

The placenta is of fetal origin and located at the interface between the mother and the fetus. The placental villous tree provides nutrition, hormones, and growth factors for fetal development. Villi are comprised of a surface layer of multinucleated syncytiotrophoblast that is bathed in maternal blood and that shares a basement membrane with a subjacent stem-cell population of mononucleated cytotrophoblasts that differentiate and fuse with the syncytium (Figure 1). Trophoblasts delimit a villous core through which the umbilical circulation is contiguous with the endothelial lining of the fetal arterial or venous vessels, including capillaries that course throughout the placental villi (Figure 1).

Differences in the expression of genes that are dependent upon fetal sex have recently been identified to influence several key signaling pathways in the placenta, including insulin-like growth factors, responses to cortisol, and placental cytokine cascades [9]. Murphy and colleagues [10] speculate that male fetuses adapt placental functions to allow for maximal fetal growth in a potentially hostile maternal allograft environment, while female fetuses reduce growth to survive such maternal influences *in vivo*. *In utero* adjustments of male and female fetuses precede observed differences in survival in the first 48 hours of preterm neonates during postnatal life: female neonates adapt better than male neonates to *ex utero* life, especially when delivered at very early gestational ages compatible with survival [11].

Sex influences on fetal programming are supported by a plethora of animal studies. For example, dietary interventions in experimental animals results in sex-specific changes in the placental methylome and transcriptome [12]–[14].

The above observations raise the theory that functional differences in gestations from male and female fetuses may be influenced by sex-biased gene expression in placental villi. Support for this theory of sex-bias in gene expression is derived from initial observations of single [9], and global [15] gene expression in biopsies of whole villi. Identification of cell types and dissection of the mechanisms involved in sex-specific regulation will likely reveal novel pathways of physiological regulation and targets for therapeutic interventions.

**Figure 2:**
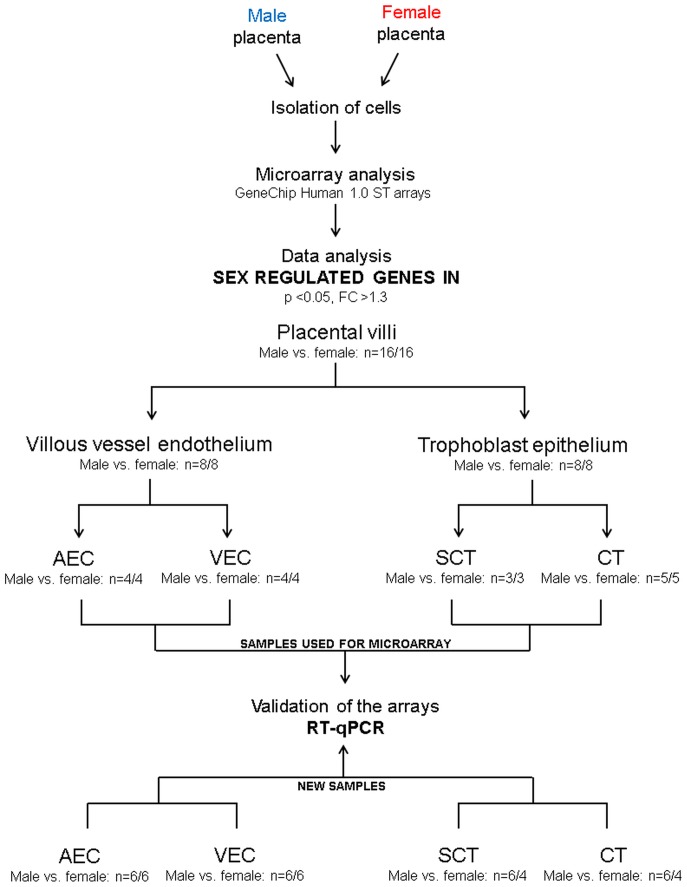
Study design. Experimental design applied to identify sex-biased gene expression in four placental cell types: syncytiotrophoblast (SCT), cytotrophoblasts (CT), arterial (AEC) and venous endothelial cells (VEC), two main placental compartments (trophoblast epithelium and villous vessel endothelium), and in the whole placental villi. The cells were isolated from placentas of male and female fetuses and their mRNA subjected to microarray analysis by hybridization to Affymetrix GeneChip Human 1.0 ST arrays. Genes that reached significance (p<0.05) and with a fold-change >1.3 were considered differentially expressed between the two sexes. Placental villi refers to the combined analysis of all expressed transcripts in syncytiotrophoblast, cytotrophoblasts, arterial and venous endothelial cells comparing gene expression in male vs. female cells. Microarray data were validated by RT-qPCR a) using identical samples as used in the microarray analysis and b) in different isolations cultured independently.

We test the hypothesis that fetal sex differentially affects gene expression in a cell-phenotype dependent manner in human placental villi. The term ‘sexome’ reflects the sum of sex-biased effects on gene networks and cell systems [16] and in this context was assessed individually in two main placental compartments, villous trophoblast epithelium and villous vessel endothelium. These compartments differ profoundly in their function. Hence, we focused on sex dependent pathways and networks rather than on individual genes. We harvested villi from placentas of human male and female newborns and cultured separately cytotrophoblasts, syncytiotrophoblast, and arterial and venous endothelial cells. All four cell types were subjected to microarray analysis by hybridization to Affymetrix GeneChip Human 1.0 ST arrays (Figure 2). We found that fetal sex does influence gene expression in a cell-type dependent manner.

## Materials and Methods

### Ethics statement

Informed written consent was obtained from the women and ethical approval was obtained from the Medical University Graz, Graz, Austria for placentas used for the isolation of endothelial cells (No.:25-008ex12/13). Placentas for trophoblast isolation were obtained by a verbal consent protocol approved by the IRB at Washington University School of Medicine, St. Louis, MO, USA. The verbal script approved by the IRB at Washington University School of Medicine, St. Louis, MO, USA is read to eligible patients undergoing a routine spontaneous or caesarian section delivery between 39–41 weeks of gestation. Following consent no further recording of the permission event is done and no medical or identification information is collected. In both cases, the sex of the infant was recorded.

### Isolation, culture and characterization of third trimester human placental trophoblast cells

Primary human villous cytotrophoblasts were isolated following digestion of placental tissue from term, uncomplicated pregnancies with trypsin-dispase-DNAse and Percoll-gradient centrifugation, as described [17]. The isolated cytotrophoblasts were cultured in a 21% O_2_/5% CO_2_ environment at 37°C in DMEM with 10% FBS, 20 mM HEPES pH 7.4, and penicillin/streptomycin on 3.5 cm diameter tissue-culture-treated plastic plates with daily media changes. To obtain cytotrophoblasts, cells were cultured for 24 h in the above medium, except using charcoal-stripped FBS, and samples were then collected. From 24–48 h of culture in this system, cytotrophoblasts spontaneously and efficiently differentiate, as indicated by greatly increased hCG and hPL secretion, and fuse, with over 80% of the nuclei present in syncytium from culture h 48–72, with the remaining present as mononucleated cells [17]. After 48 h, culture medium was changed to media with charcoal-stripped FBS and syncytiotrophoblast samples were harvested after 72 h of culture.

### Isolation, culture and characterization of third trimester human placental EC

Primary arterial and venous endothelial cells were isolated following a standard protocol [18] from third trimester human placentas after uncomplicated pregnancy and vaginal or caesarean section delivery. Endothelial cells were characterized by a rigorous immuno-cytochemical analysis and internalization of acetylated low-density-lipoprotein (Biomedical Technologies, Stoughton MA) [19]. Only paired arterial and venous endothelial cells originating from the same placenta were used for microarray analysis. Prior to microarray analysis 800,000 cells were seeded on 1% (v/v) gelatin-coated flasks (75 cm^2^) using Endothelial Basal Medium (EBM, Cambrex, Clonetics, USA) supplemented with the EGM-MV BulletKit (Clonetics). Endothelial cells were cultured for 48 h at 21% O_2_/5% CO_2_ environment at 37°C.

### RNA isolation and validation

Total RNA from the pelleted cells was isolated and purified with RNeasy mini Kit (Qiagen, Hilden, Germany). RNA was tested for quality using a BioAnalyzer BA2100 (Agilent, Foster City, CA, USA) with the RNA 6000 Nano LabChip Kit (Agilent, Foster City, CA, USA). Only samples with a RIN (RNA Integrity Number) ≥8.5 were used for further analyses.

### Sex confirmation in cytotrophoblast and arterial endothelial cells

Because syncytiotrophoblast samples derived by fusion from cytotrophoblasts and venous endothelial cells were isolated from the same placenta as the arterial cells, fetal sex was confirmed only in one cell type per layer. Sex of arterial endothelial cells was confirmed by testing the presence or absence of the *SRY* gene (sex-determining region Y) [20] on the Y chromosome using GeneAmp PCR Kit from Invitrogen. Genomic DNA from each sample was isolated with the QIAamp DNA Mini Kit (Qiagen, Hilden, Germany). PCR was performed using Mastercycler (Eppendorf, USA) for 27 cycles at 94°C for 30 s (denature), 58°C for 30 s (anneal) and for 72°C for 1 min (extension) with an initial denaturation step for 15 min at 95°C.

Sex of the cytotrophoblasts was confirmed by assaying expression of the *DDX3Y* gene (DEAD (Asp-Glu-Ala-Asp) box polypeptide 3, Y-linked) on the Y chromosome [21] by RT-PCR using One-step Kit (Qiagen).

Specific primers for *SRY* (forward 5′-CTCCGGAGAAGCTCTTCCTT-3′ and reverse 5′-CAGCTGCTTGCTGATCTCTG-3′) and *DDX3Y* gene (forward 5′- ATTGGCAATCGTGAAAGACC-3′ and reverse 5′- TACTGCCGGTTGCCTCTACT-3′) were purchased from Ingenetix (Austria). PCR products were separated by electrophoresis through 2% (wt/vol) agarose gel, stained with ethidium bromide and visualized under UV light.

### Array hybridization and scanning and data pre-processing methods

Total RNA was labeled using Ambion WT Expression Kit for Affymetrix GeneChip Whole transcript (WT) Expression Arrays (Life Technologies; Carlsbad, CA, USA). Hybridization of all samples to GeneChip Human 1.0 ST arrays was performed according to the manufacturer's instructions (Affymetrix, Santa Clara, CA, USA). For each sample, 200 ng of total RNA were reverse transcribed to double stranded cDNA. During the second cycle of the cDNA synthesis, single-stranded cDNA was generated. Washing and staining (GeneChip® HT hybridization, Wash and Stain Kit, Affymetrix, Santa Clara, CA, USA) was done with the Affymetrix Genechip® fluidics station 450. Arrays were scanned with the Affymetrix GeneChip scanner GCS3000. Labeling controls and hybridization controls were evaluated with Expression Console EC 1.1.

Hybridization and data pre-processing were carried out at the Division Core Facility for Molecular Biology at the Centre of Medical Research at the Medical University of Graz, Graz, Austria. Microarray data were analyzed with RMA (robust multi-chip average) - including background correction, quantile normalization across all arrays, log2 transformation, and median polish summarization using Genomic Suite v6.5 (Partek Inc, St Louis, MO, USA) [22]. Hierarchical clustering analysis and Principal Component Analysis were performed to compare the global expression profile for each sample and to check for outliers. All samples passed the quality control check and no outliers were detected.

### Biostatistical analysis of microarray data

In order to identify differentially expressed genes influenced by sex in each of the four different placental cell types, ie. syncytiotrophoblast, cytotrophoblast, arterial and venous endothelial cells, we performed one-way ANOVA for each cell type comparing male vs. female using Partek Genomics Suite v6.5 platform (Partek GS, Partek Inc., Revision 6.5). Furthermore, for the analysis of sex-biased gene expression in trophoblast epithelium (comprising syncytiotrophoblast and cytotrophoblasts), villous vessel endothelium (comprising arterial and venous endothelial cells) and placental villi (comprising syncytiotrophoblast, cytotrophoblasts as well as arterial and venous endothelial cells) (Figure 2) a 2-way ANOVA model was used. In this model the cell type was included as variable in addition to fetal sex. All analyses were followed by Fisher's post-hoc test. P-values for the influence of cell type on gene expression in the respective placental compartments studied can be found in supporting information Table S2A. P-values were adjusted for multiple hypothesis testing based on the false discovery rate (FDR) by the Benjamini-Hochberg method (R/Bioconductor package ‘multtest’). Probes showing p-value <0.05 (or where indicated FDR <5%), fold-change >1.3, and annotated as RefSeq transcript/gene (NM_) were considered differentially expressed and further analyzed.

To reveal the importance and function of the sex-biased genes in trophoblast epithelium and villous vessel endothelium, we visualized gene expression levels (log2 fold changes) as heatmaps using Genesis [23] and investigated affected biological pathways and networks with Ingenuity Pathway Analysis (IPA) (Ingenuity® Systems, Ink, Redwood City, CA, USA), Pathway Studio® (Ariadne Genomics, Ink, Rockville, MD, USA) [24]. Gene Ontology analysis was performed using the Database for Annotation, Visualization and Integrated Discovery (DAVID) [25], Pathway Studio® and Panther [26]. Distribution of gene expression (log2(M/F)) were generated using (Gaussian) kernel density estimation within R.

The microarray data have been deposited in NCBI's Gene Expression Omnibus following MIAME guidelines and are accessible through GEO Series accession number GSE44368.

### Microarray validation by reverse transcription-qPCR (RT-qPCR)

Microarray data were validated by RT-qPCR a) using identical samples as used in the microarray analysis and b) in different isolations cultured independently (Figure 2.).The cDNA was synthesized from 500 ng total RNA according to the manufacturer's instructions (SuperScript II Reverse Transcriptase protocol from Invitrogen, USA). Real-time was performed with TaqMan gene expression assays from Applied Biosystems (CA, USA) for the respective genes and the ABI Prism 5,700 Sequence Detection System. Hypoxanthine-guanine phosphoribosyltransferase *(HPRT1*) was used as a reference gene [27], [28] since it did not vary between male and female groups in this study (Table S1). The amplification efficiency of four randomly selected TaqMan gene expression assays *DDX3Y*, *TFPI2*, *FGF5* and *ERAP2* ranged from 90–95% and for *HPRT1* the efficiency was 93%. Amplification efficiency was tested according to manufacturer's (Applied Biosystems, CA, USA) instructions “Guide to Performing Relative Quantitation of Gene Expression Using Real-Time Quantitative PCR”. Data were analyzed according to the 2^-ΔΔCt^ method [29]. For statistical analysis a student t-test was performed using SigmaPlot software package.

## Results

### Influence of fetal sex on gene expression in four villous cell phenotypes

**Figure 3:**
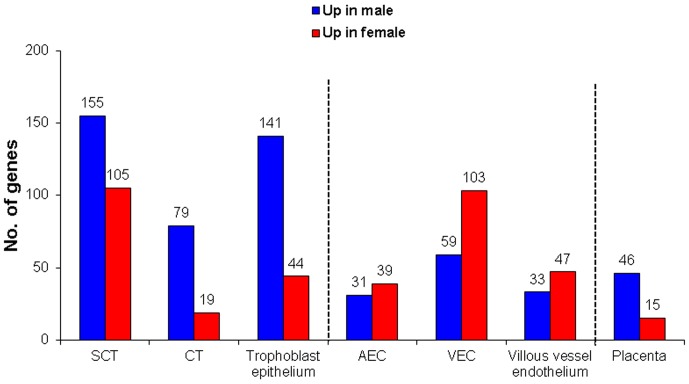
Number of genes showing sex-biased expression in four distinct placenta cell types. Number of differentially expressed genes (p<0.05, fold-change >1.3) between male and female syncytiotrophoblasts, cytotrophoblasts, arterial and venous endothelial cells, trophoblast epithelium, villous vessel endothelium, and for placental villi is shown as number of genes upregulated in males vs. the number of genes upregulated in females. Placental villi refers to combined analysis of all expressed transcripts in syncytiotrophoblasts, cytotrophoblasts, arterial and venous endothelial cells.

**Figure 4:**
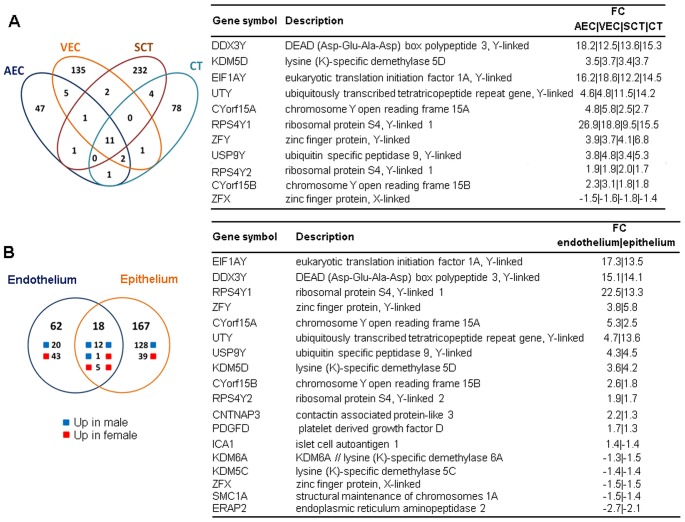
Genes whose expression is regulated by fetal sex in common to all cell phenotypes. Venn diagram depicting the number of sex regulated genes shared between (A) syncytiotrophoblast (SCT), cytotrophoblast (CT), arterial (AEC) and venous endothelial cells (VEC) (B) endothelial and epithelial compartment. Genes found to be in common are shown in boxes on the right (A and B).

Whole genome microarray analysis revealed that sex-biased gene expression varied among placental cell types (Figure 3, Figure S1B). Genes that reached the significance level of p<0.05 and with a fold-change >1.3 were analyzed further. Only 1.3% of all annotated transcripts were differentially expressed between the two sexes in arterial, 2.4% in venous endothelial cells, 6.3% in syncytiotrophoblasts, and 1.5% in cytotrophoblasts, respectively. When combining the data sets of all four placental cell phenotypes and removing duplicates, fetal sex influenced the expression of 9% of all genes (p<0.05, fold-change >1.3). In cytotrophoblasts and syncytiotrophoblast more genes showed higher expression when derived from pregnancies with male fetuses than when derived from pregnancies with female fetuses, opposite to arterial and venous endothelial cells that had more genes with higher expression if the fetus was a female (Figure 3). Eleven genes differentially expressed between males and females were in common for all cell types. Ten of these eleven genes were Y-linked and thus expressed at higher levels in male cells, while one was X-linked and expressed at higher levels in female cells (Figure 4A). Top 10 genes with higher expression in male and 10 with higher expression in female placental cell types are listed in Table 1.

**Table 1:** Top 10 genes showing higher expression in male and top 10 with higher expression in female placental cell types.

Cell type	SCT			CT			AEC			VEC		
	Gene symbol	p-value	FC	Gene symbol	p-value	FC	Gene symbol	p-value	FC	Gene symbol	p-value	FC
1	RBMY2EP	<0.001	23.83	RPS4Y1	<0.001	15.50	RPS4Y1	<0.001	26.89	RPS4Y1	<0.001	18.81
2	RBMY1A1	0.001	14.47	DDX3Y	<0.001	15.26	DDX3Y	<0.001	18.21	EIF1AY	<0.001	18.62
3	DDX3Y	<0.001	13.60	EIF1AY	<0.001	14.53	EIF1AY	<0.001	16.16	DDX3Y	<0.001	12.50
4	EIF1AY	<0.001	12.15	UTY	<0.001	14.16	NLGN4Y	<0.001	11.05	NLGN4Y	<0.001	7.36
5	UTY	<0.001	11.54	ZFY	<0.001	6.78	CYorf15A	<0.001	4.82	CYorf15A	<0.001	5.79
6	RBMY1B	0.001	9.96	USP9Y	<0.001	5.28	UTY	<0.001	4.63	USP9Y	<0.001	4.80
7	RBMY1A1	0.001	9.95	KDM5D	<0.001	4.70	ZFY	<0.001	3.86	UTY	<0.001	4.78
8	RPS4Y1	<0.001	9.49	VNN2	0.040	2.81	USP9Y	<0.001	3.82	MPZL2	0.047	3.92
9	RBMY1A1	<0.001	6.46	CYorf15A	0.004	2.69	GSTT1	0.033	3.76	KDM5D	<0.001	3.72
10	ERAP2	<0.001	5.64	KRT6A	0.043	2.39	RGS5	0.023	3.75	ZFY	<0.001	3.69
11	SPATA5L1	0.046	−1.75	SNORD80	0.013	−1.49	PRKX	0.001	−1.62	LAMA2	0.017	−2.03
12	ZFX	0.010	−1.76	BLID	0.021	−1.50	STEAP2	0.045	−1.66	NNAT	0.009	−2.18
13	SCARNA9L	0.008	−1.77	FLJ43390	0.037	−1.51	MYOZ2	0.008	−1.68	JMY	0.006	−2.26
14	SGK269	0.040	−1.78	KDM6A	<0.001	−1.53	PARD6G	0.024	−1.91	SYT14	0.038	−2.30
15	SNORD25	0.037	−1.80	RGS2	0.012	−1.55	C21orf94	0.006	−1.99	CHSY3	0.024	−2.30
16	SNORD116-24	0.037	−1.84	ABCC2	0.016	−1.55	TFPI2	0.042	−2.24	FAM26E	0.030	−2.43
17	PARVA	0.029	−1.94	SLC13A4	0.048	−1.64	GSTT2	0.032	−2.35	ITGA4	0.047	−2.55
18	ZNF674	0.004	−2.05	RNU5B-1	0.049	−1.92	MIR217	0.034	−2.55	LPHN2	0.017	−2.63
19	UQCRH	0.012	−2.10	SNORD3A	0.010	−1.93	C21orf94	0.005	−3.98	FGF5	0.032	−2.72
20	AQPEP	0.045	−2.24	SCARNA9L	0.004	−2.08	F2RL2	0.009	−4.92	NRK	0.005	−4.73

FC =  fold-change is the ratio of mean expression for male vs. female cells. SCT =  syncytiotrophoblasts, CT =  cytotrophoblasts, AEC =  arterial endothelial cells and VEC =  venous endothelial cells.

Global gene expression patterns were visualized with the principal component analysis that revealed a clear separation according to cell type, but not by sex (Figure S1A). This was confirmed with hierarchical clustering analysis that showed the longest distance between trophoblast epithelium and villous vessel endothelium, followed by shorter distances between syncytiotrophoblasts and cytotrophoblasts, arterial and venous endothelial cells and the least distance between the two sexes (not shown). Nevertheless, the global microarray analysis revealed the average sex specific difference in gene expression ranged from 19% in cytotrophoblasts to 32% in syncytiotrophoblast. (Figure S1C). Hence, sex-biased gene expression varied between the cell types: Cytotrophoblasts had the highest proportion of genes showing no sex bias in expression, whereas syncytiotrophoblast revealed the highest sex differences in expression (Figure S1B).

Genes with a different degree of sex bias, based on fold-change, were selected for validation by RT-qPCR (Table 2). All selected genes, except *ANGPT1*, were confirmed to be significantly changed in arterial and venous endothelial cells. *KRT6A* in syncytiotrophoblast, and *KTR6A* and *PCD11X* in cytotrophoblasts did not reach statistical significance between the sexes, albeit average fold-changes were comparable between RT-qPCR and microarray analysis.

**Table 2:** Validation of microarray data with RT-qPCR.

Method		Microarray				RT-PCR			
Cell type		SCT		CT		SCT		CT	
Statistical parameters		p-value	FC	p-value	FC	p-value	FC	p-value	FC
**Y-linked genes**	**DDX3Y**	<0.001	13.60	<0.001	15.26	expressed only in male cells			
	**UTY**	<0.001	11.54	<0.001	14.16	expressed only in male cells			
	**ZFY**	<0.001	4.06	<0.001	6.78	expressed only in male cells			
	**KDM5D**	0.001	3.39	<0.001	4.70	expressed only in male cells			
**X-linked genes**	**PCDH11X**	N.C.		0.001	1.78	N.T.		0.063	2.7
	KDM6A*	N.C.		<0.001	−1.53	N.T.		0.046	−1.90
**Autosomal genes**	**IDO1**	N.C.		0.011	2.10	N.T.		0.008	2.98
	**ERAP2**	<0.001	5.64	N.C.		0.001	22.81	N.T.	
	LAMA4*	<0.001	1.45	N.C.		0.049	1.28	N.T.	
	PLCB4*	0.002	1.35	N.C.		0.006	9.86	N.T.	
	ERAP2*	<0.001	5.64	N.C.		0.019	5.52	N.T.	
	ADA*	N.C.		0.004	1.73	N.T.		0.022	4.91
	**KRT6A**	0.014	1.22	0.043	2.39	0.310	1.48	0.090	1.15
	CCL4*	N.C.		0.030	1.81	N.T.		0.026	7.66

Method		Microarray				RT-PCR			
Cell type		AEC		VEC		AEC		VEC	
Statistical parameters		p-value	FC	p-value	FC	p-value	FC	p-value	FC
**Y-linked genes**	**DDX3Y**	<0.001	18.21	<0.001	12.50	expressed only in male cells			
	**UTY**	<0.001	4.63	<0.001	4.78	expressed only in male cells			
	**ZFY**	<0.001	3.86	<0.001	3.69	expressed only in male cells			
	**KDM5D**	<0.001	3.49	<0.001	3.72	expressed only in male cells			
**Autosomal genes**	**F2RL2**	0.009	−4.92	N.C.		0.002	−7.69	N.T.	
	PLSCR4*	0.008	1.75	N.C.		0.046	1.84	N.T.	
	TFPI2*	0.045	−2.24	N.C.		0.028	−2.36	N.T.	
	XAF1*	0.02	1.42	N.C.		0.060	2.39	N.T.	
	PC*	N.C.		0.04	1.45	N.T.		0.01	1.38
	FGF5*	N.C.		0.03	−2.72	N.T.		0.03	−4.72
	ANGPT1*	N.C.		0.02	−1.33	N.T.		0.81	−3.33

FC =  fold-change is the ratio of mean expression for male vs. female cells. N. C. =  no change. N.T. =  not tested. SCT =  syncytiotrophoblasts, CT =  cytotrophoblasts, AEC =  arterial endothelial cells and VEC =  venous endothelial cells. * =  genes validated using samples distinct from those used in microarray analysis.

Imprinted genes are known to regulate placental and fetal growth. In order to investigate whether they account for the observed sexual dimorphism in gene expression among placental cell types, the lists of differentially expressed genes were compared with an online catalogue of imprinted genes (www.geneimprint.com). From 63 recognized imprinted genes three matched to our list of genes. These were *ANKRD11* (Ankyrin repeat domain 11) in cytotrophoblasts (fold-change = −1.4), *NNAT* (Neuronatin) in arterial (fold change = −2.2) and *MEST* (Mesoderm specific transcript homolog) in venous endothelial cells (fold change = 2.7).

### Sex dependent gene expression profile differences in trophoblast epithelium vs. villous vessel endothelium

In the trophoblast epithelium, 185 genes showed sex-biased expression vs. 80 genes in the villous vessel endothelium (Figure 3). Not only was the number of genes showing sexual dimorphic expression different between the two placental compartments, but also the direction of gene regulation was different. More female upregulated genes were found in the villous vessel endothelium, whereas trophoblast epithelium showed increased number of transcripts when derived from pregnancies with male fetuses. From the total of 185 sex-biased genes in trophoblast epithelium and villous vessel endothelium, 18 genes were in common for the two villous compartments. Among these, 12 were upregulated in males, five in females and one had a discordant expression in epithelium vs. endothelium (Figure 4B). Illustration of these 185 genes is shown by a heat map (Figure S2).

**Figure 5:**
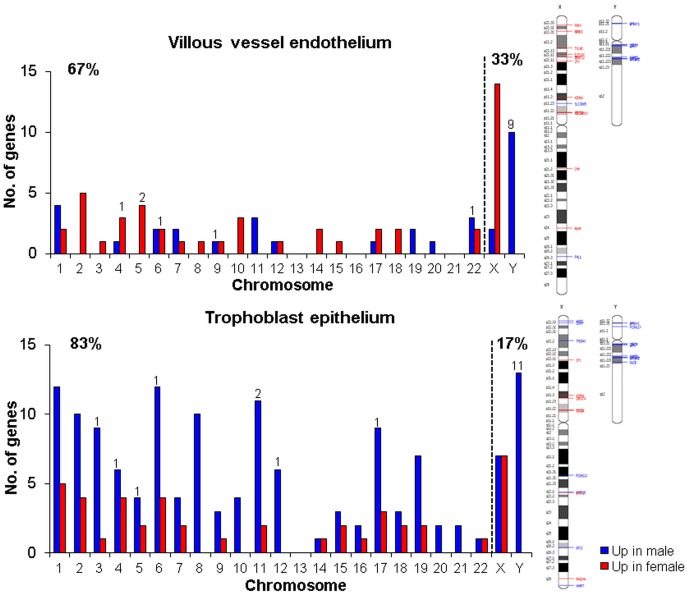
Chromosomal distribution of genes showing sex-biased expression in placental endothelium and epithelium. The number of male- and female-biased genes (p<0.05, fold-change >1.3) is shown for each chromosome separately. The number on top of the bars indicates the number of genes with fold-change >2. The proportion of genes located on autosomal vs. sex chromosomes is given on the top right and left of each graphs, respectively. An ideogram at the right depicts the specific location of upregulated genes in males (blue line) and females (red line) on X and Y chromosomes.

Chromosomal distribution of male and female genes that were differentially expressed in trophoblast epithelium vs. villous vessel endothelium was analyzed to identify a preferential clustering of the sex-biased genes to a distinct chromosome (Figure 5). In endothelium the differentially regulated genes were distributed across all chromosomes, although, not surprisingly, chromosome X had the highest number of female-biased genes. Only three chromosomes (13, 16 and 21) did not harbor transcripts with sex bias. In the trophoblast epithelium, male upregulated genes were clearly overrepresented on all chromosomes, except for chromosome 14, where female upregulated genes prevailed, and for chromosome 22, which had an equal number of male- and female-biased genes. Among the 80 and 185 sex biased genes in the villous vessel endothelium and trophoblast epithelium, the majority, i.e. 66% and 85%, respectively, were located on autosomal chromosomes. Only 26 and 27 genes were located on sex chromosomes in the villous vessel endothelium and trophoblast epithelium (Figure 5, Table 3.). The exact genomic location and the respective fold-change for each gene are listed in Table S2.

**Table 3:** Distribution and the level of sex- biased gene expression across autosomal and sex chromosomes in placental endothelium vs. epithelium.

	Autosomal chromosomes			Sex chromosomes		
Placental compartment	No. of genes (p<0.05, FC>1.3)	% of genes	Average level of global sex difference (%)	No. of genes (p<0.05, FC>1.3)	% of genes	Average level of global sex difference (%)
**Villous vessel endothelium**	54	67	17	26	33	99
**Trophoblast epithelium**	158	83	24	27	17	114

% of genes is given in relation to the total number of genes showing sex biased expression in villous vessel endothelium and trophoblast epithelium, respectively. The average level of global sex differences is calculated across all genes showing significant change in expression (p<0.05) on autosomal vs. sex chromosomes for each placental compartment separately.

Global X-chromosome gene expression prevailed in both compartments of females, with endothelium showing fewer genes but with greater male vs. female differences than the epithelium (Figure S3). Expression of Y-linked genes was limited to males, as expected, but the distribution of the relative expression for endothelium and epithelium was similar to the X-chromosome.

### Sex regulated genes in villous vessel endothelium and trophoblast epithelium – implications for biological relevance

*Biological processes*: Male-biased genes in the trophoblast epithelium were overrepresented in the clusters of genes involved in cell adhesion, and responses to stimuli, including inflammatory and immune responses. Remarkably, none of these processes were linked to female-biased genes in the trophoblast epithelium. Sex-biased genes related to cell adhesion and responses to stimuli were also noted in the villous vessel endothelium, where an additional pathway involving glutathione metabolism was also differentially influenced by fetal sex (Table S3, S4 and S5).

*Molecular functions*: Male-biased genes in the trophoblast epithelium were associated with oxygen binding and oxygen transporter activity, as well as hydrolase, metallopeptidase, oxydoreductase and MHC class II receptor related activities. Female-biased genes were related only to ion binding. Genes upregulated in villous vessel endothelium from males were linked to glutathione transferase, estradiol 17-beta-dehydrogenase and RNA and rRNA binding activities, while actin binding, transferase and dioxygenase activities were enriched with female upregulated genes (Table S6 and S7).

*Metabolic and signaling pathways*: Fetal sex influenced gene enrichment for networks related to cellular movement, development, lipid metabolism, cell death, and antigen presentation in trophoblast epithelium (Table S8). Networks in the endothelial compartment amongst others also included genes important for cellular movement and cell trafficking (Table S8). Notably, one network differentially regulated by fetal sex in the villous vessel endothelium (Figure S4A) showed direct or indirect associations of different molecules with the regulator *NFκB*. A specific network of genes markedly enriched in the trophoblast epithelium was one showing interactions with TNF-alpha (Figure S4B).

In the trophoblast epithelium, the highest enrichment score was for genes in i.e. atherosclerosis and graft-versus-host disease signaling pathways (Table 4) which parallel the identified sex regulated biological processes. The most significant canonical pathways that were different between the sexes of the villous vessel endothelium included genes involved in glutathione, androgen and estrogen metabolism (Table 4). We also aimed to identify upstream transcriptional regulators that could influence differential gene expression among cells from the two sexes. Surprisingly, none of the known upstream regulators reached the activation, or inhibition, threshold z-score in the endothelium. In contrast, fifteen regulators were identified as important for the sex-biased gene expression in the trophoblast epithelium (Table S9 and Figure S5), with eleven regulators predicted to be activated.

**Table 4:** Top 5 significantly enriched canonical pathways in Ingenuity Pathway Analysis.

Placental compartment	Top canonical pathways	Molecules	%
**Villous vessel endothelium**	Glutathione Metabolism	GSTT2/GSTT2B, GSTT1, GGT5	6.0
	Androgen and Estrogen Metabolism	HSD17B10, STS, HSD17B14	4.3
	Estrogen-Dependent Breast Cancer Signaling	HSD17B10, HSD17B14	3.1
	IL-10 Signaling	HMOX1, IL1A	2.8
	Regulation of eIF4 and p70S6K Signaling	EIF1AY, EIF1AX, RPS4Y2, RPS4Y1	2.5
**Trophoblast epithelium**	Airway Pathology in Chronic Obstructive Pulmonary Disease	IL8, MMP9, MMP1	37.5
	Atherosclerosis Signaling	ALOX5, IPOD, IL8	9.4
	Graft-versus-Host Disease Signaling	CD86, HLA-DQA1, IL1RA	8.7
	Arginine and Proline Metabolism	ABP1, VNN2, VNN1	5.0
	Role of Macrophages, Fibroblasts and Endothelial Cells in Rheumatoid Arthritis	CAMK2D, FRZB, FZD5, IL8, MMP1, MMP3, PDGFD, PLCB4, SFRP, TGFB1	3.5

The proportion (%) refers to the amount of sex-biased genes found to play a role in the respective pathway.

### Pathways in placental villi subjected to sex influence

We identified differentially expressed transcripts in all four cell types combined, to gain insights into overall sex-biased gene expression in placental villi. The expression of 61 genes, constituting <1% of all annotated genes, differed between the two fetal sexes (Figure 3). Among these, 46 were upregulated in males and 15 in females, as illustrated by the heat map in Figure S6. Because too few transcripts showed a fold-change >1.3, we used the unfiltered list of genes with significance of p<0.05. Metabolic and signaling pathways in the placenta that were most affected by fetal sex included insulin action, B cell activation, mitochondrial protein transport, Notch-MEF/MYOD, PDGFR-STAT and ActivinR-SMAD2/3 signaling, histone ubiquitylation and regulation of the cell cycle (Table S10).

## Discussion

Earlier studies on sexual dimorphism in mammals used RNA isolated from whole tissues, which included placental villi [15], [30]. Our study extends these findings to identify sex effects on gene expression in four distinct placental cell types. Moreover, our data are the first to compare global gene expression by microarray analysis in isolated, characterized somatic cells from the same tissue, human placental villi. The data show that fetal sex differentially affects gene expression in a cell-phenotype dependent manner in human placental villi, and fetal sex influences gene expression among all four cell phenotypes studied: cytotrophoblasts and syncytiotrophoblast epithelial cells and arterial and venous endothelial cells.

Stark and colleagues [11], [31] showed that the enzymatic activity of placental 11-beta hydroxysteroid dehydrogenase 2 differs between male and female fetuses, framing potential differences in steroid levels and stress responses in the two sexes. We build on these reports to show there is a disparity in sex biased gene expression in two of the main functional components of placental villi, villous trophoblast epithelium and the villous core endothelium. Hence, we focused our analysis on identifying key pathways, biological processes and networks subject to fetal sex influences with the aim to explain reported differences in placental function and disparity in pregnancy outcome. This approach of using genomic variation, even more modest that here, and sets of functionally related genes to analyze regulated pathways and networks is not novel [32], [33]. The pathways we have identified are the overlapping results of different software packages used for gene ontology analysis, making our findings robust.

The key findings of our study are: (i) all four cell types analyzed *in vitro* varied in the extent of sex-biased gene expression, despite the fact that these cells originate from the same organ; (ii) transcripts of male fetuses prevailed in the epithelial compartment, represented by cytotrophoblasts and syncytiotrophoblasts, whereas the endothelial compartment, represented by arterial and venous endothelial cells, showed more female-biased genes; (iii) sex-biased genes in both the epithelial and endothelial compartments clustered with groups of genes linked to distinct biological functions and molecular pathways.

The overlapping biological processes especially influenced by sex-bias were inflammatory and immune response, cell adhesion and responses to stimuli in the epithelium, while glutathione metabolic processes and responses to stimuli were overrepresented in the endothelium. We conclude that fetal sex biases gene expression in human placental cells based on cell type and tissue compartment studied. We speculate that such sex-biased gene expression contributes to the differential responses of male and female offspring to developmental and environmental modulators to yield part of the different outcomes known to occur in male and female newborns based on sex.

Although sexual dimorphism in mammals correlates with hormonal differences, hormones are not the only determinants of such dimorphism. Before gonad differentiation occurs, male and female bovine preimplantation embryos display phenotypic differences that can only be due to the differences in sex chromosome dosage [34]. Male and female cells from mice isolated prior to sexual differentiation respond differently to exogenous stressors *in vitro* [35]. As these responses cannot be attributed to sex hormone differences inherent *in vivo*, they raise the question about the mechanism of the disparate response. Sex chromosome transcripts are differentially expressed between the sexes and are proposed to regulate the transcription of autosomal genes and, therefore, to program the cells to respond in a sex-dependent manner [36].

The transcriptome from intact human placental villi is also characterized by sexual dimorphism, which is not restricted to X- and Y-linked genes [15]. We herein extend the previous studies by showing that the transcriptome of human placental syncytiotrophoblast, cytotrophoblasts, arterial and venous endothelial cells depends on the sex of the fetus.

Compared to the female, the male sex is a risk factor for adverse pregnancy outcome, with pregnancies with male fetuses exhibiting a higher incidence of gestational diabetes, preterm birth, premature preterm rupture of membranes, and macrosomia (reviewed in [5], [37]). The reason remains to be elucidated as to why male fetuses are more vulnerable (or why the females are less susceptible) for such complications while *in utero*.

Like Sood and coauthors [15], we note that the sex-biased gene expression is distributed across all chromosomes. Their majority is on the autosomal chromosomes and only 33% and 17% of the sex-biased genes in the villous vessel endothelium and in the trophoblast epithelium, respectively, is on sex chromosomes. Male-specific, Y-linked genes in the fetus may pose a special challenge to mothers during pregnancy. Several genes on the Y-chromosome encode epitopes that contribute to HY antigen, a group of male-specific peptides acting as minor histocompatibility antigens (reviewed in [38]). We found some of these genes *(DDX3Y*, *UTY*, *KDM5D*, *USP9Y*, *EIF1AY* and *RPS4Y1*) are predominantly expressed in males in both villous trophoblast and endothelium. These epitopes associate with sub-optimal pregnancy outcome, because miscarriages are more frequent after a firstborn male [39]. The presence or absence of HY antigens is also associated with transplantation success, as kidneys from male donors have an increased rate of rejection in female hosts compared to kidneys received from female donors [40]. Notably, Ingenuity Pathway Analysis identified that one of the markedly enriched pathways in our data was graft-versus-host disease signaling.

Male-biased genes in the trophoblast compartment are also markedly enriched in genes related to the immune and inflammatory system, which further supports the hypothesis of reduced maternal-fetal compatibility for male fetuses. These genes included *HLA-DQB*1 (syncytiotrophoblast), *HLA-DQA1* (syncytiotrophoblast and cytotrophoblast), *HCP5* (cytotrophoblast), *NOS1* (cytotrophoblast), *FSTL3*, *PAPPA*, *SPARCL* and *FCGR2C* (trophoblast epithelium), *CD34* (cytotrophoblast), *HLA-F* (cytotrophoblast), *BCL2* (syncytiotrophoblast). These and other immune-related genes were also found in a heterogeneous cell isolate prepared from placental villous mesenchyme [15]. We speculate that differences between our study results and those presented by Sood and colleagues [15] relate to expression profiles from stromal cells and macrophages present in the Sood study but absent from our more purified isolates. These findings parallel the hypothesis of increased vulnerability *in utero* of male fetuses related to sex differences of the feto-placental immune system in relation to preterm delivery [41]. Placentas from male fetuses showed more prominent evidence of chronic inflammation and more advanced histopathological lesions associated with this inflammatory response, especially at the basal plate interface.

Our study has limitations, despite careful planning. Firstly, the cytotrophoblasts and syncytiotrophoblasts studied *in vitro* and used for the microarray analysis necessarily originated from different placentas than the specimens from which the arterial and venous endothelial cell phenotypes were derived. Different procedures were needed to isolate the epithelial and endothelial cell types and the bulk of the placenta was required for a given tissue isolate. Importantly, we controlled for this approach as a potential source of variance by including the patient as one random variable in the statistical analysis. Trophoblasts required *in vitro* culture (though not cell division) while both endothelial phenotypes required *in vitro* propagation to obtain enough cells for the microarray analysis. Thus, we acknowledge our study uses models, not *in vivo* exposed specimens, where results may be different.

Our data, combined with emerging evidence on the influence of fetal sex on placental development and function [9], [13] offer a partial explanation for the increased risk for preterm birth and premature preterm rupture of membranes in pregnancies carrying male fetuses. The maternal immune responses are critical to the success of the allograft of pregnancy. We predict that the sex differences in placental gene expression, specifically related increased male expression of immune and inflammatory genes and genes involved in graft-versus-host disease, contribute a sex-biased profile of placental gene expression that contributes to differential outcomes for male and female newborns after exposure to clinical entities such as preterm birth or premature rupture of membranes. Furthermore, we identified a sex bias in genes important for cellular metabolism and cellular responses to injury in the placental villous cell phenotypes studied. Therefore, our results highlight the need to consider fetal sex as an independent variable in studies of placental villi and cell culture experiments of villous components.

## Supporting Information

Figure S1 **Influence of sex and cell type on global gene expression in four placental cell types.** Principal component analysis plot of all samples (A). Histogram showing male (M): female (F) ratios of expression of all transcripts in syncytiotrophoblast (SCT), cytotrophoblasts (CT), arterial (AEC) and venous endothelial cells (VEC), respectively. Sample aggregation is based on their similarity. The average sex difference of gene expression levels is shown for each cell type separately. The proportion was calculated over all genes that showed significant (p<0.05) expression changes between the two sexes.
(TIF)

Figure S2 **Sex-biased genes in villous vessel endothelium and trophoblast epithelium.** Heat map illustration of differentially expressed genes between males and females in villous vessel endothelium (CT-SCT) and trophoblast epithelium (AEC-VEC). The color scale goes from blue to red, representing genes upregulated in males (blue) and females (red), respectively.
(TIF)

Figure S3 **Histograms depicting male (M):female (F) ratios of global gene expression.** Histograms are constructed for villous vessel endothelium and trophoblast epithelium for transcripts located on X- and Y-chromosome, respectively.
(TIF)

Figure S4 **Pathway networks for (A) villous vessel endothelium and (B) trophoblast epithelium.** Networks depict most significantly enriched pathways for (A) Villous vessel endothelium: Cellular movement, hematological system development and function, immune cell trafficking and (B) villous vessel endothelium: Cell death, cell-to-cell signaling and interaction, antigen presentation. Networks were constructed using differentially expressed genes between male and female placental compartments (p<0.05, fold-change >1.3) with Ingenuity Pathway Analysis. Male-biased genes are shown in green while female-biased genes in red.
(TIF)

Figure S5 **Upstream regulators.** Schematic representation of the upstream regulators for genes differentially regulated by fetal sex in trophoblast epithelium (p<0.05, FC >1.3). IL1RN was the only upstream regulator that showed sex-bias. No upstream regulator has been identified for sex-biased genes in the endothelium.
(TIF)

Figure S6 **Heat map illustration of the sex-biased genes in the human placental villi.** Placental villi refers to the combined analysis of all expressed transcripts in syncytiotrophoblast, cytotrophoblasts, arterial and venous endothelial cells comparing gene expression in male vs. female cells. The color scale ranges from blue to red, representing genes upregulated in males and females, respectively.
(TIF)

Table S1 **Mean Ct values for the reference gene hypoxanthine-guanine phosphoribosyltransferase (** ***HPRT1*** **) for male and female group of cells for each cell type.**
(DOCX)

Table S2 **Genomic location of genes showing sex biased expression in trophoblast epithelium and villous vessel endothelium (A) and in their component cells syncytiotrophoblast (SCT) (B), cytotrophoblasts (CT) (C), arterial (AEC) (D) and venous endothelial cells (VEC) (E).**
(XLSX)

Table S3 **Biological processes displaying sex bias in villous vessel endothelium and trophoblast epithelium (Panther).**
(DOCX)

Table S4 **Biological processes displaying sex bias in villous vessel endothelium and trophoblast epithelium (Pathway studio).**
(DOCX)

Table S5 **Biological processes displaying sex bias in villous vessel endothelium and trophoblast epithelium (DAVID).**
(DOCX)

Table S6 **Molecular functions displaying sex bias in endothelium and epithelium (Pathway studio).**
(XLS)

Table S7 **Molecular functions displaying sex bias in endothelium and epithelium (DAVID).**
(DOCX)

Table S8 **Top networks generated using Ingenuity Pathway Analysis with the highest enrichment score of the differentially expressed genes (p<0.05, FC >1.3).**
(DOCX)

Table S9 **List of regulators whose downstream target genes are differentially expressed between male and female trophoblast epithelium identified using Ingenuity Pathway Analysis.**
(DOCX)

Table S10 **Metabolic and signaling pathways identified by Pathway studio for placental villi.**
(DOCX)
