# Supplementary figures and images for: The Human Placental Sexome Differs between Trophoblast Epithelium and Villous Vessel Endothelium
Source: PLoS One. 2013 Oct 29;8(10):e79233. doi: 10.1371/journal.pone.0079233 (PMC3812163; doi:10.1371/journal.pone.0079233)

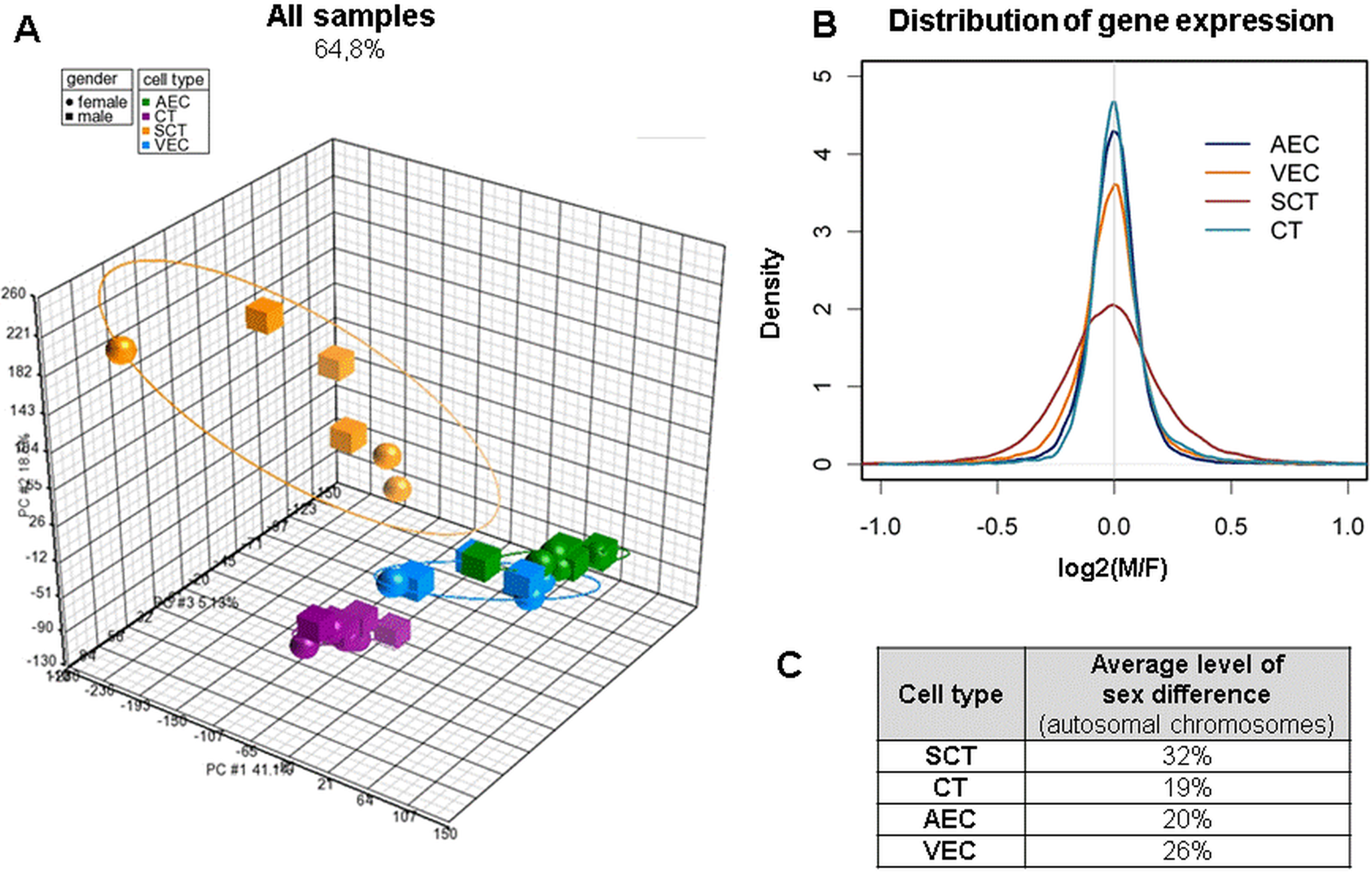

Supplement: Figure S1 — Influence of sex and cell type on global gene expression in four placental cell types. Principal component analysis plot of all samples (A). Histogram showing male (M): female (F) ratios of expression of all transcripts in syncytiotrophoblast (SCT), cytotrophoblasts (CT), arterial (AEC) and venous endothelial cells (VEC), respectively. Sample aggregation is based on their similarity. The average sex difference of gene expression levels is shown for each cell type separately. The proportion was calculated over all genes that showed significant (p<0.05) expression changes between the two sexes. (TIF) [file pone.0079233.s001.tif]

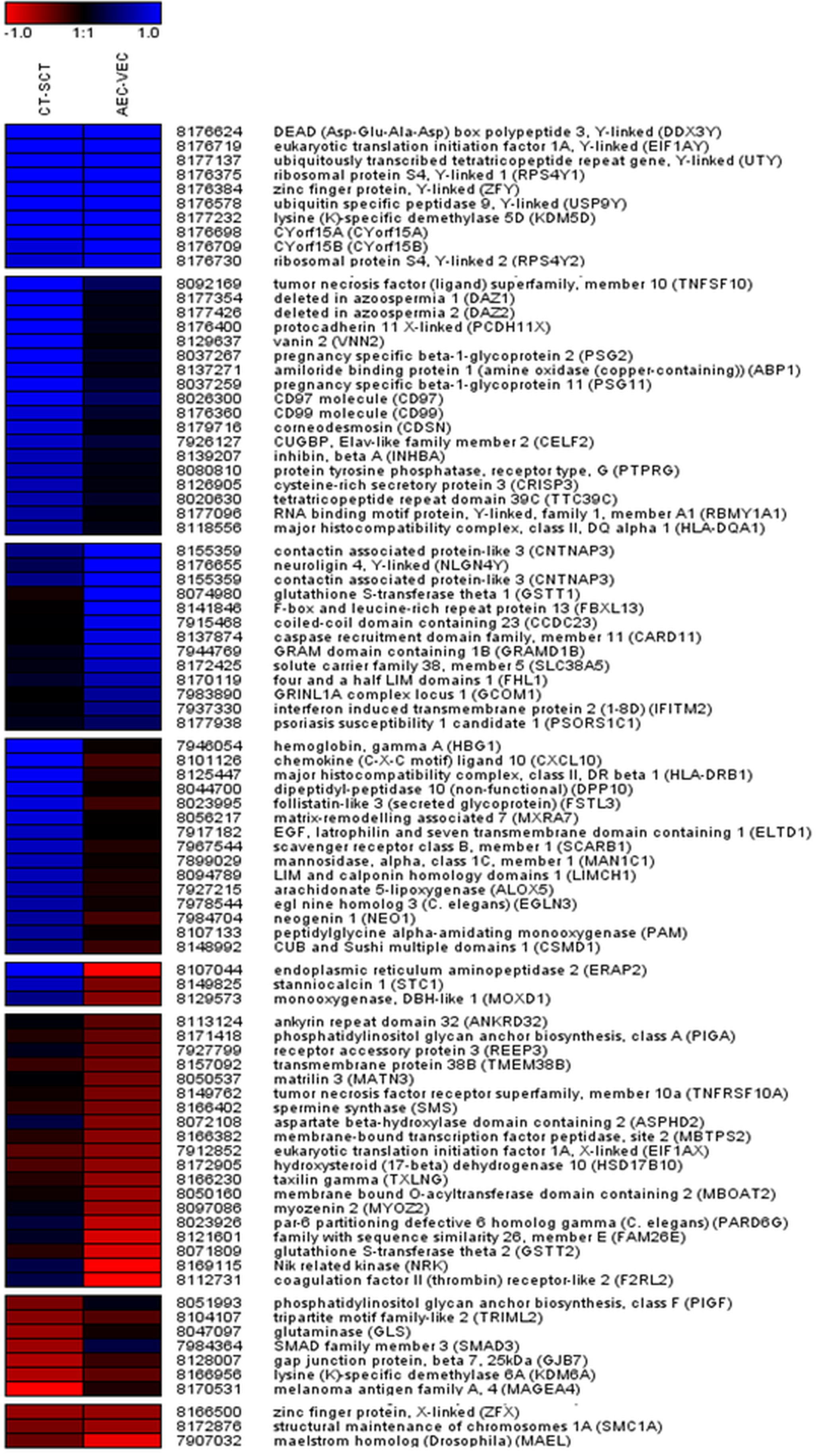

Supplement: Figure S2 — Sex-biased genes in villous vessel endothelium and trophoblast epithelium. Heat map illustration of differentially expressed genes between males and females in villous vessel endothelium (CT-SCT) and trophoblast epithelium (AEC-VEC). The color scale goes from blue to red, representing genes upregulated in males (blue) and females (red), respectively. (TIF) [file pone.0079233.s002.tif]

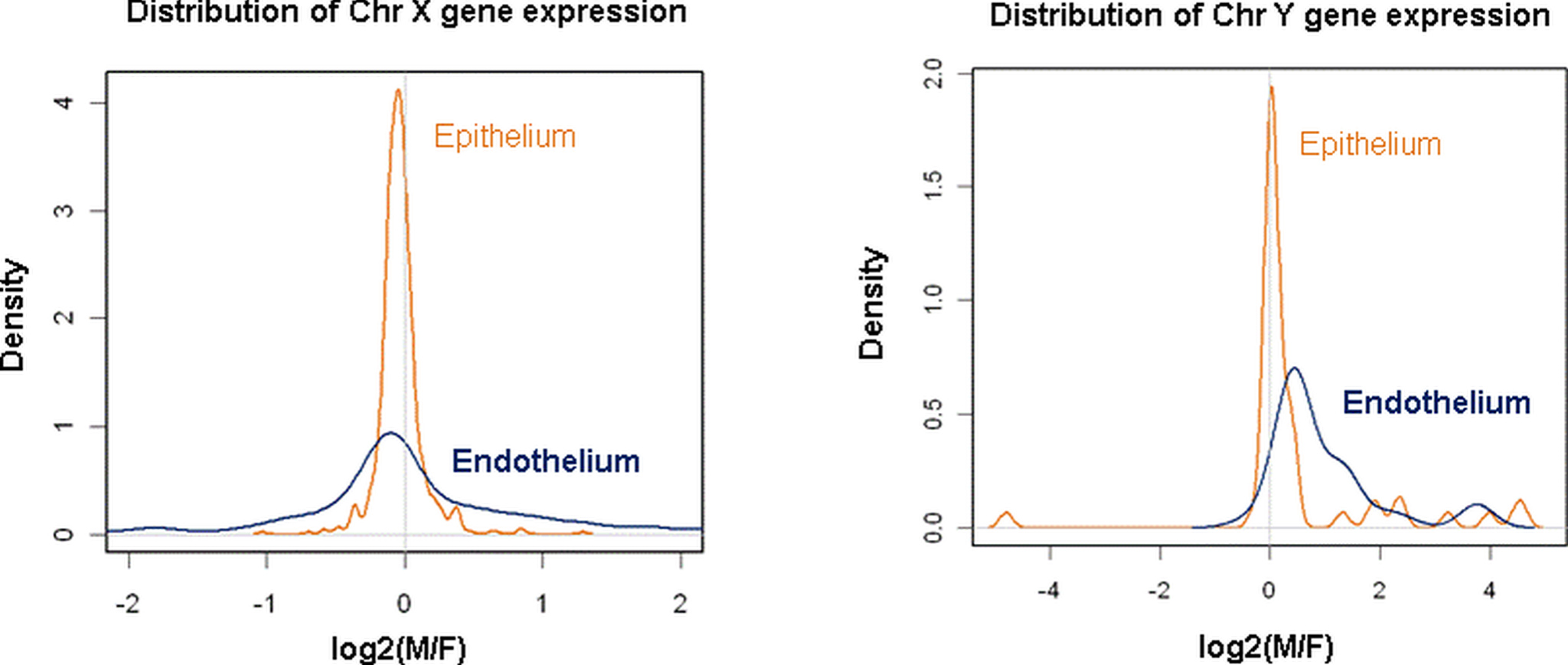

Supplement: Figure S3 — Histograms depicting male (M):female (F) ratios of global gene expression. Histograms are constructed for villous vessel endothelium and trophoblast epithelium for transcripts located on X- and Y-chromosome, respectively. (TIF) [file pone.0079233.s003.tif]

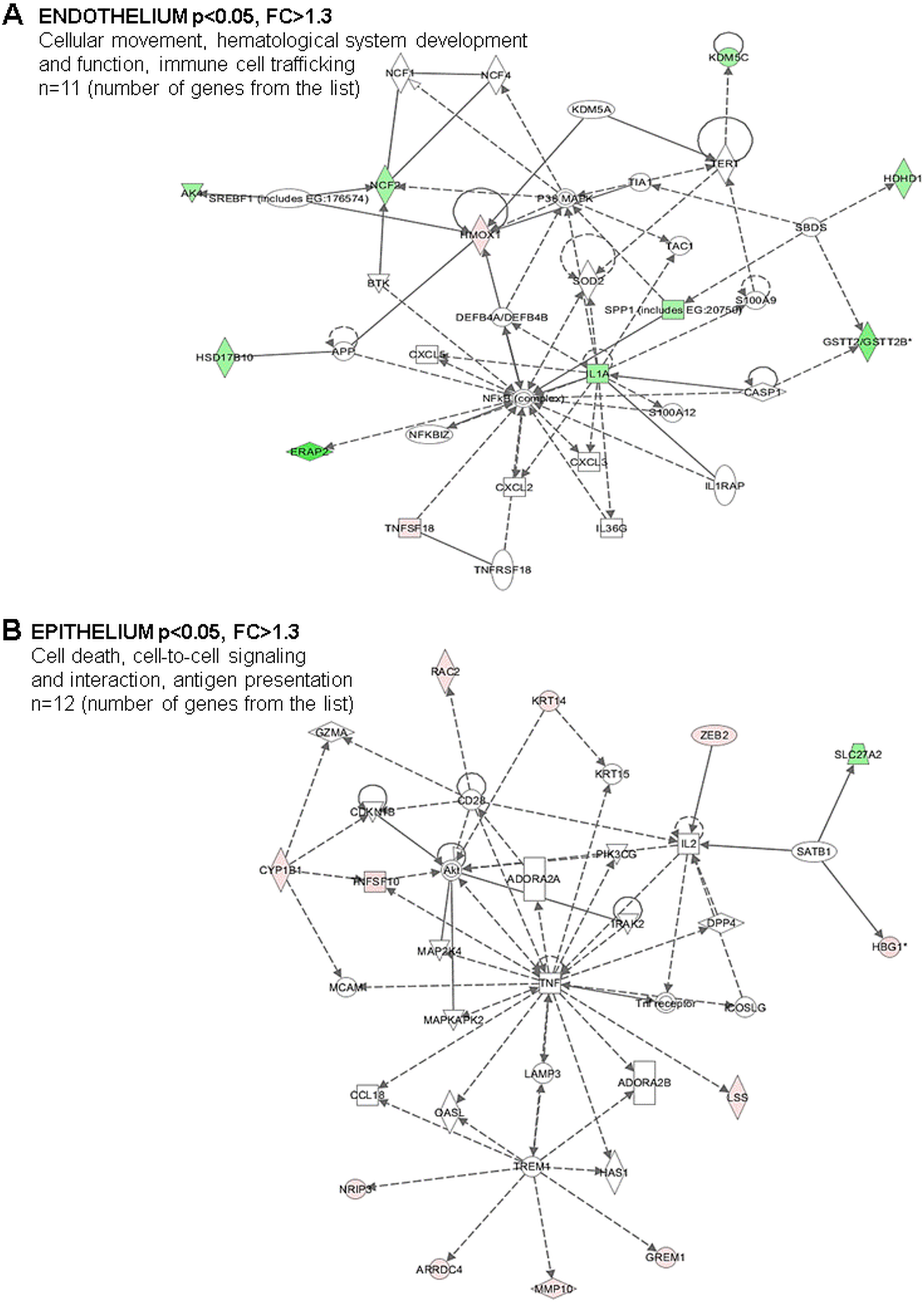

Supplement: Figure S4 — Pathway networks for (A) villous vessel endothelium and (B) trophoblast epithelium. Networks depict most significantly enriched pathways for (A) Villous vessel endothelium: Cellular movement, hematological system development and function, immune cell trafficking and (B) villous vessel endothelium: Cell death, cell-to-cell signaling and interaction, antigen presentation. Networks were constructed using differentially expressed genes between male and female placental compartments (p<0.05, fold-change >1.3) with Ingenuity Pathway Analysis. Male-biased genes are shown in green while female-biased genes in red. (TIF) [file pone.0079233.s004.tif]

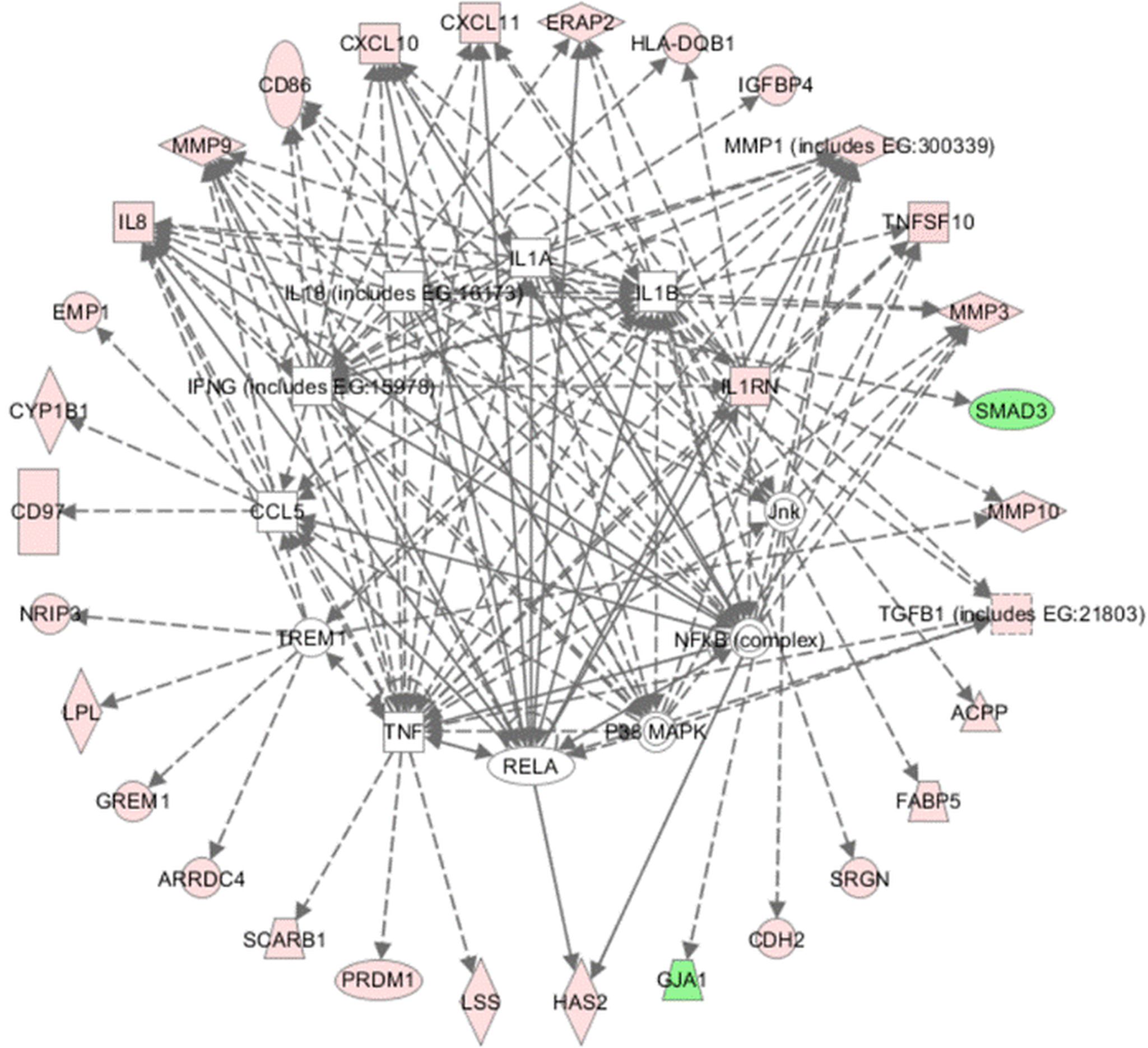

Supplement: Figure S5 — Upstream regulators. Schematic representation of the upstream regulators for genes differentially regulated by fetal sex in trophoblast epithelium (p<0.05, FC >1.3). IL1RN was the only upstream regulator that showed sex-bias. No upstream regulator has been identified for sex-biased genes in the endothelium. (TIF) [file pone.0079233.s005.tif]

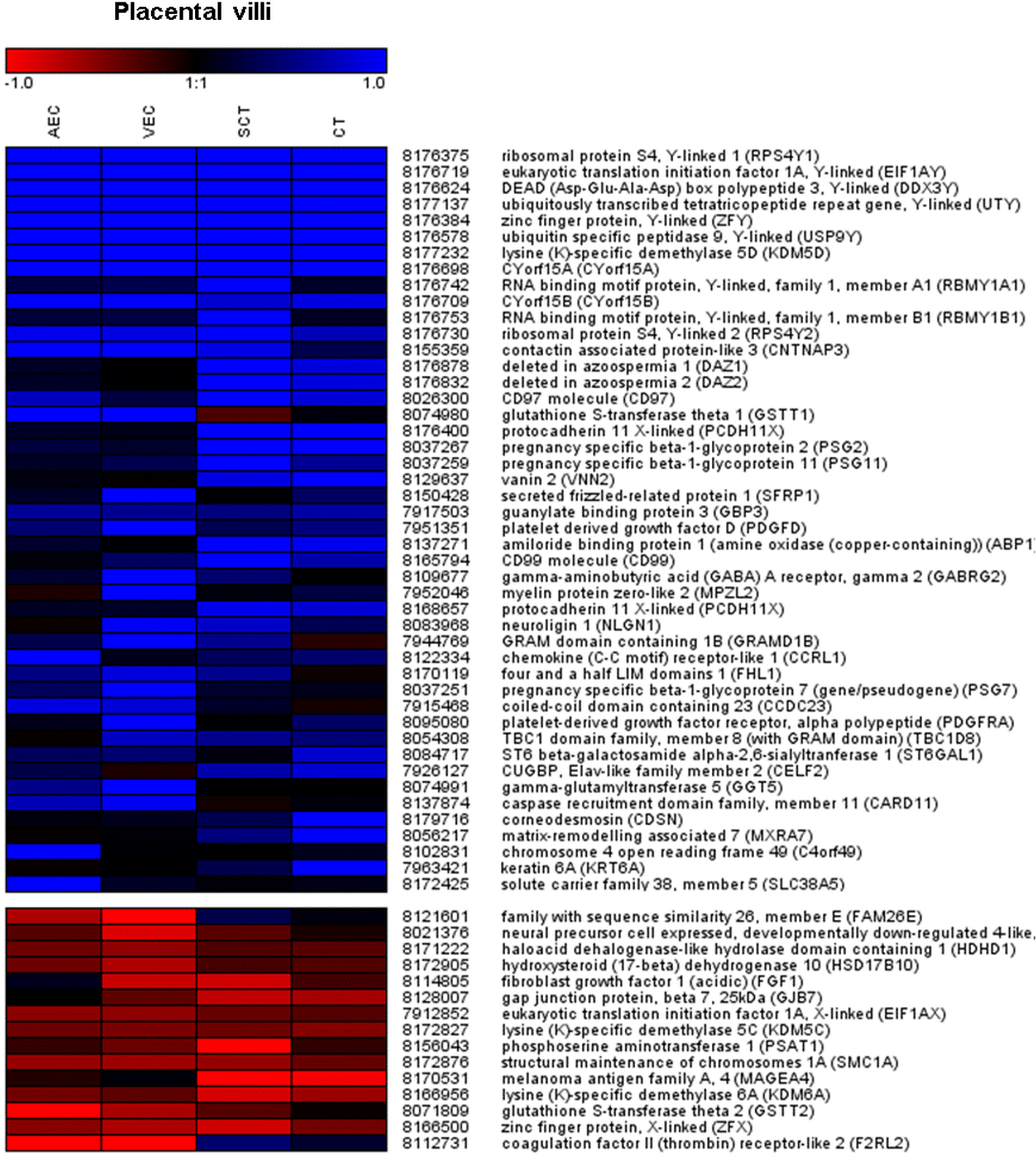

Supplement: Figure S6 — Heat map illustration of the sex-biased genes in the human placental villi. Placental villi refers to the combined analysis of all expressed transcripts in syncytiotrophoblast, cytotrophoblasts, arterial and venous endothelial cells comparing gene expression in male vs. female cells. The color scale ranges from blue to red, representing genes upregulated in males and females, respectively. (TIF) [file pone.0079233.s006.tif]
