# Supplementary material for: The Human Placental Sexome Differs between Trophoblast Epithelium and Villous Vessel Endothelium
Source: PLoS One. 2013 Oct 29;8(10):e79233. doi: 10.1371/journal.pone.0079233 (PMC3812163; doi:10.1371/journal.pone.0079233)
Supplement: Table S1 — Mean Ct values for the reference gene hypoxanthine-guanine phosphoribosyltransferase ( HPRT1 ) for male and female group of cells for each cell type. (DOCX) [file pone.0079233.s007.docx]

|  | **Male cells** | | **Female cells** | | **p-value** |
| --- | --- | --- | --- | --- | --- |
|  | **Mean Ct** | **SD** | **Mean Ct** | **SD** |  |
| **SCT** | 28.93 | 0.71 | 28.70 | 0.40 | 0.56 |
| **CT** | 29.11 | 0.67 | 29.61 | 0.38 | 0.20 |
| **AEC** | 25.78 | 0.37 | 25.87 | 0.38 | 0.66 |
| **VEC** | 25.58 | 0.39 | 25.77 | 0.29 | 0.30 |

**Table S1. Mean Ct values for the reference gene hypoxanthine-guanine phosphoribosyltransferase (HPRT1) for male and female group of cells for each cell type.**

HPRT1 was used as a reference gene in RT-qPCR for data analysis using the 2^-ΔΔCt^ method [29]. SD = standard deviation. SCT = syncytiotrophoblasts, CT = cytotrophoblasts, AEC = arterial endothelial cells and VEC = venous endothelial cells.
