# Supplementary material for: The Human Placental Sexome Differs between Trophoblast Epithelium and Villous Vessel Endothelium
Source: PLoS One. 2013 Oct 29;8(10):e79233. doi: 10.1371/journal.pone.0079233 (PMC3812163; doi:10.1371/journal.pone.0079233)
Supplement: Table S3 — Biological processes displaying sex bias in villous vessel endothelium and trophoblast epithelium (Panther). (DOCX) [file pone.0079233.s009.docx]

**Table S3. Biological processes displaying sex bias in villous vessel endothelium and trophoblast epithelium (Panther).**

| **Compartment** | **Biological process** | **%** | **p-value** |
| --- | --- | --- | --- |
| Villous vessel endothelium | Response to stimulus | 1.4 | 8E-04 |
|  | Immune system process | 1.1 | 3E-03 |
| Trophoblast epithelium | Immune system process | 2.4 | 9E-07 |
|  | Cell adhesion | 2.7 | 4E-04 |
|  | Cell communication | 1.6 | 7E-04 |
|  | Signal transduction | 1.6 | 7E-04 |
|  | Response to stimulus | 2.3 | 1E-03 |
|  | Cell-cell adhesion | 3.4 | 2E-03 |
|  | Macrophage activation | 5.4 | 4E-03 |
|  | Cell-cell signaling | 2.4 | 4E-03 |
|  | Immune response | 2.7 | 6E-03 |

Only genes with FC >1.3 were used. Significance level was set to p <0.05 for both, genes and processes. The significance level for biological processes was tested using Fishers Exact test. FC = fold-change is the ratio of mean expression for male vs. female cells. The proportion (%) refers to the amount of sex-biased genes found to play a role in the respective biological process.
