# Supplementary material for: The Human Placental Sexome Differs between Trophoblast Epithelium and Villous Vessel Endothelium
Source: PLoS One. 2013 Oct 29;8(10):e79233. doi: 10.1371/journal.pone.0079233 (PMC3812163; doi:10.1371/journal.pone.0079233)
Supplement: Table S4 — Biological processes displaying sex bias in villous vessel endothelium and trophoblast epithelium (Pathway studio). (DOCX) [file pone.0079233.s010.docx]

**Table S4. Biological processes displaying sex bias in villous vessel endothelium and trophoblast epithelium (Pathway studio).**

| **Compartment** | **Biological process** | **Genes** | **p-value** |
| --- | --- | --- | --- |
| Villous vessel endothelium | Response to organic cyclic compound | STS,IL1A,MET,NCF2,TNFRSF10A, GSTT1,SRR,AK4 | 5.0E-08 |
|  | Glutathione metabolic process | GSTT1,GSTT2,GGT5 | 1.2E-04 |
|  | Chromatin modification | UIMC1,KDM6A,KDM5C,KDM5D,UTY | 2.9E-04 |
|  | Steroid catabolic process | STS,HSD17B14 | 3.1E-04 |
|  | Positive regulation of I-kappaB kinase-NF-kappaB cascade | HMOX1,IL1A,TNFRSF10A,CARD11 | 3.4E-04 |
|  | Response to organic substance | SPP1,IL1A,TNFRSF10A,AK4 | 4.7E-04 |
|  | Positive regulation of interleukin-2 biosynthetic process | IL1A,CARD11 | 5.0E-04 |
|  | Oxidation-reduction process | ASPHD2,HMOX1,HSD17B10,KDM6A, KDM5C,KDM5D,UTY,HSD17B14 | 5.2E-04 |
|  | Response to lipopolysaccharide | IL1A,NCF2,CSF2RB,SRR | 8.9E-04 |
|  | Cytokine-mediated signaling pathway | IL1A,OAS1,CSF2RB,IFITM2 | 1.6E-03 |
| Trophoblast epithelium | Inflammatory response | TGFB1,IL8,ALOX5,CXCL10,IL1RN,CXCL11,LYZ,IGFBP4,PLA2G7,NOX4,A2M, CD97,VNN1 | 2.3E-08 |
|  | Pregnancy | TGFB1,IL1RN,MMP3,PAPPA,SFRP4, A2M,PSG11,PSG2 | 4.0E-07 |
|  | Cell adhesion | CNTNAP3,CDH2,SCARB1,NEO1, TGFBI,CD84,CD99,CD97,NLGN1, CDSN,EMR2,CD96,PCDH11X, PCDH11Y,PCDH18 | 5.9E-06 |
|  | Immune response | IL8,TNFSF10,CXCL10,IL1RN,CXCL11,SMAD3, CD86, CD97, HLA-DQB1,HLA-DQA1, COLEC12,CD96,HLA-DQA2 | 6.6E-06 |
|  | Response to glucocorticoid stimulus | IL1RN,PAPPA,SFRP4,FABP4,A2M, PAM,CTSL2 | 9.4E-06 |
|  | Collagen catabolic process | MMP9,MMP1,MMP3,MMP10 | 1.4E-05 |
|  | Hemopoietic progenitor cell differentiation | TGFB1,PLEK,FSTL3 | 2.6E-05 |
|  | Regulation of Rab GTPase activity | TBC1D3B,TBC1D3G,TBC1D3H, TBC1D3F,TBC1D8 | 3.1E-05 |
|  | Lipid metabolic process | IL1RN,SCARB1,LPL,PLA2G7,APOD, FABP4,PLCB4,FABP5,SLC27A2,NAAA | 4.1E-05 |
|  | Positive regulation of apoptosis | TGFB1,TNFSF10,MMP9,NOX4,TXNIP,SFRP4,ID3,FRZB | 4.4E-05 |

Only genes with FC >1.3 were used. Significance level was set to p <0.05 for both genes and processes. Significance level for biological processes was tested using Fishers Exact test. FC = fold-change is the ratio of mean expression for male vs. female cells.
