# Supplementary material for: The Human Placental Sexome Differs between Trophoblast Epithelium and Villous Vessel Endothelium
Source: PLoS One. 2013 Oct 29;8(10):e79233. doi: 10.1371/journal.pone.0079233 (PMC3812163; doi:10.1371/journal.pone.0079233)
Supplement: Table S5 — Biological processes displaying sex bias in villous vessel endothelium and trophoblast epithelium (DAVID). (DOCX) [file pone.0079233.s011.docx]

**Table S5. Biological processes displaying sex bias in villous vessel endothelium and trophoblast epithelium (DAVID).**

| **Compartment** | **Expression** | **Biological process** | **Score** | **%** | **FDR [%]** | **GENES** |
| --- | --- | --- | --- | --- | --- | --- |
| Villous vessel endothelium | Up in male | Cofactor metabolic process | 195 | 9 | 46 | GGT5,HMOX1,GSTT1 |
|  |  | Glutathione metabolic process | 28 | 6 | 49 | GGT5, GSTT1 |
|  | Up in female | Anatomical structure development | 2527 | 26 | 44 | GMFB,HSD17B10,MATN3, STS,CAP2,MET,CHM, TMOD2, SRR, KIAA1217, SPP1 |
| Trophoblast epithelium | Up in male | Multicellular organismal process | 4280 | 37 | 4 | OR4F21, MMP9, PRRX1, ZEB2, MMP3, CDSN, TGFB1, MMP1, CXCL10, OR4C13, GPC3, TGFBI, CNTNAP3, ERAP2, HTR1F, KDM5D, OR4F16, MMP10, KRT14, RPS4Y1, STC1, ADAM19, PRDM1, EMP1, CYP1B1, TBC1D8, USP9Y, CDH2, BICC1, GREM1, RAC2, CAMK2D, SCARB1, HBB, SRGN, TXNIP, DAZ1, DAZ2, OR2A4, PLEK, OR2A7, IL8, NLGN1, CRYZ, FRZB, SNAI2, PCDH18, SFRP4, FABP4, HTR2B, IGFBP4, FABP5 |
|  |  | Response to stimulus | 3502 | 31 | 7 | HLA-DQB1, A2M, CYP1B1, OR4F21, MRE11A, CXCL11, MMP3, TGFB1, CXCL10, CD97, CD96, OR4C13, RAC2, TGFBI, CAMK2D, VNN1, SCARB1, ERAP2, TXNIP, CRISP3, OR2A4, GBP5, PLEK, OR2A7, IL8, IL1RN, LYZ, OR4F16, SNAI2, HLA-DQA2, HLA-DQA1, CD84, CD86, TNFSF10, VAMP7, LILRB4, KRT14, PLA2G7, FABP4, STC1, ALOX5, GBP4, IGFBP4 |
|  |  | Positive regulation of biological process | 2033 | 19 | 40 | TBC1D8, MMP9, KLRK1, PRRX1, ZEB2, TGFB1, CXCL10, GPC3, RAC2, CAMK2D, VNN1, SCARB1,  PDGFD, HBB, TXNIP, LPL, DAZ1, DAZ2, IL8, PLEK, CD86, TNFSF10, FABP4, ALOX5, PRDM1, HTR2B |
|  |  | Immune system process | 998 | 16 | 0.06 | HLA-DQB1, CRISP3, GBP5, IL8, PLEK, MMP9, IL1RN, KLRK1, CXCL11, HLA-DQA2, HLA-DQA1, TGFB1, CXCL10, CD97, CD96, TNFSF10, CD86, VAMP7, LILRB4, VNN1, ERAP2, GBP4 |
|  |  | Immune response | 690 | 14 | 0.01 | HLA-DQB1, CRISP3, GBP5, IL8, IL1RN, CXCL11, HLA-DQA2, HLA-DQA1, TGFB1, CXCL10, CD97, CD96, TNFSF10, CD86, VAMP7, LILRB4, VNN1, ERAP2, GBP4 |
|  |  | Response to chemical stimulus | 1281 | 13 | 56 | TXNIP, A2M, CYP1B1, IL8, IL1RN, MMP3, CXCL11, TGFB1, CXCL10, RAC2, KRT14, CAMK2D, FABP4, VNN1, SCARB1, STC1, ALOX5 |
|  |  | Cell adhesion | 700 | 12 | 0.74 | PLEK, PCDH11Y, PCDH11X, NLGN1, CD99, CDH2, NEO1, CDSN, PCDH18, CD97, CD84, CD96, TGFBI,  CNTNAP3, VNN1, SCARB1 |
|  |  | Defense response | 615 | 10 | 2 | CRISP3, A2M, IL8, IL1RN, LYZ, CXCL11, TGFB1, CXCL10, CD97, CD84, PLA2G7, VNN1, ALOX5, IGFBP4 |
|  |  | Response to wounding | 530 | 10 | 0.5 | A2M, IL8, PLEK, IL1RN, LYZ, CXCL11, TGFB1, CXCL10, CD97, PLA2G7, VNN1, SCARB1, ALOX5, IGFBP4 |
|  |  | Regulation of signal transduction | 878 | 10 | 55 | TBC1D3F, TBC1D8, TBC1D3G, PLEK, TBC1D3H, PRRX1, FSTL3, ZEB2, CDH2, GREM1, FRZB, TGFB1, TBC1D3B, TNFSF10, GPC3, HTR2B, TBC1D3 |
|  |  | Tissue development | 665 | 9 | 25 | TXNIP, GPC3, KRT14, PRRX1, ZEB2, GREM1, SNAI2, CDSN, EMP1, FABP5, TGFB1, SRGN |
|  |  | Inflammatory response | 325 | 9 | 0.096 | CD97, A2M, IL8, IL1RN, LYZ, PLA2G7, VNN1, ALOX5, CXCL11, TGFB1, IGFBP4, CXCL10 |
|  |  | Cell motion | 475 | 7 | 46 | CD97, IL8, PSG2, VNN1, ZEB2, SCARB1, CDH2, VNN2, TGFB1 |
|  |  | Regulation of response to stimulus | 465 | 7 | 42 | A2M, CD86, PLEK, IL8, KLRK1, FABP4, ZEB2, GREM1, TGFB1 |
|  |  | Cell proliferation | 436 | 7 | 33 | CD86, TGFBI, ZEB2, MAPRE2, SCARB1, PSPH, EMP1, TGFB1, IGFBP4 |
|  | Up in female | - | - | - | - | - |

Only genes with FC >1.3 were used. Significance level was set to p <0.05 for both genes and processes. FC = fold-change is the ratio of mean expression for male vs. female cells; score = number of genes after enrichment involved in the respective biological process and related to genes that show sex-biased expression. The proportion (%) refers to the amount of sex-biased genes found to play a role in the respective biological process. FDR = false discovery rate.
