# Supplementary material for: The Human Placental Sexome Differs between Trophoblast Epithelium and Villous Vessel Endothelium
Source: PLoS One. 2013 Oct 29;8(10):e79233. doi: 10.1371/journal.pone.0079233 (PMC3812163; doi:10.1371/journal.pone.0079233)
Supplement: Table S7 — Molecular functions displaying sex bias in endothelium and epithelium (DAVID). (DOCX) [file pone.0079233.s013.docx]

**Table S7. Molecular functions displaying sex bias in endothelium and epithelium (DAVID).**

| **Compartment** | **Expression** | **Molecular function** | **Score** | **%** | **FDR [%]** | **GENES** |
| --- | --- | --- | --- | --- | --- | --- |
| Villous vessel endothelium | Up in male | RNA binding | 718 | 15 | 25 | RPS4Y2, DDX3Y, EIF1AY, RPS4Y1, OAS1 |
|  |  | rRNA binding | 29 | 6 | 41 | RPS4Y2, RPS4Y1 |
|  | Up in female | transferase activity | 49 | 7 | 8 | CHM, GSTT2, SMS |
|  |  | actin binding | 326 | 12 | 10 | GMFB, CAP2, XIRP2, TMOD2, JMY |
|  |  | dioxygenase activity | 66 | 7 | 13 | KDM6A, ASPHD2, KDM5C |
| Trophoblast epithelium | Up in male | metallopeptidase activity | 183 | 5 | 3 | MMP10, PAPPA, MMP9, ERAP2, ADAM19, MMP3, MMP1 |
|  |  | oxygen binding | 43 | 3 | 5 | CYP1B1, HBG1, HBA2, HBA1, HBB |
|  |  | oxygen transporter activity | 13 | 2 | 5 | HBG1, HBA2, HBA1, HBB |
|  |  | metalloendopeptidase activity | 104 | 4 | 10 | MMP10, MMP9, ADAM19, MMP3, MMP1 |
|  |  | peptidase activity, acting on L-amino acid peptides | 549 | 8 | 10 | NRIP3, MMP10, CTSL2, USP9Y, PAPPA, MMP9, DPP10, ERAP2, ADAM19, MMP3, MMP1 |
|  |  | calcium ion binding | 919 | 11 | 10 | SPARCL1, PCDH11Y, MMP9, PCDH11X, CDH2, MMP3, MMP1, PCDH18, CD97, ABP1, MMP10, PLCB4, ELTD1, EMR2, ALOX5 |
|  |  | MHC class II receptor activity | 19 | 2 | 11 | HLA-DQB1, HLA-DQA2, HLA-DQA1 |
|  |  | peptidase activity | 574 | 8 | 13 | NRIP3, MMP10, CTSL2, USP9Y, PAPPA, MMP9, DPP10, ERAP2, ADAM19, MMP3, MMP1 |
|  |  | cytokine activity | 195 | 4 | 18 | TNFSF10, IL8, IL1RN, CXCL11, GREM1, CXCL10 |
|  |  | pantetheine hydrolase activity | 3 | 1 | 26 | VNN1, VNN2 |
|  |  | hydrolase activity | 2283 | 19 | 30 | CTSL2, USP9Y, MRE11A, MMP9, DPP10, MMP3, PSPH, MMP1, PLCB4, RAC2, PAPPA, DDX3Y, VNN1, ERAP2, VNN2, PTPRB, NRIP3, LPL, GBP5, PTPRG, LYZ, ACPP, MMP10, PLA2G7, ADAM19, GBP4 |
|  |  | iron ion binding | 308 | 5 | 31 | CYP1B1, UTY, HBG1, HBA2, ALOX5, HBA1, HBB, KDM5D |
|  |  | receptor binding | 886 | 10 | 34 | LPL, IL8, IL1RN, KLRK1, CXCL11, GREM1, TGFB1, CXCL10, TNFSF10, TGFBI, CNTNAP3, STC1, PDGFD |
|  |  | protein binding | 8154 | 53 | 42 | A2M, MMP9, PRRX1, FSTL3, ZEB2, PSPH, CXCL11, CDSN, TGFB1, CXCL10, CD97, CD96, PLCB4, GPC3, APOD, EIF1AY, TGFBI, CNTNAP3, ELTD1, PDGFD, DOCK10, KDM5D, PTPRG, PCDH11Y, PCDH11X, LRRC1, LYZ, CD84, CD86, VAMP7, KRT14, CELF2, HBG1, MAPRE2, STC1, TMSB4X, ADAM19, CTSL2, PAM, CYP1B1, USP9Y, MRE11A, KLRK1, CDH2, NEO1, GREM1, CBR1, RAC2, CAMK2D, SCARB1, HBB, SRGN, PTPRB, TXNIP, LPL, DAZ1, DAZ2, PLEK, IL8, IL1RN, NLGN1, CD99, HBA2, HBA1, FRZB, CAMK2N1, PCDH18, TNFSF10, SFRP4, FABP4, ALOX5, FABP5, IGFBP4 |
|  |  | oxidoreductase activity | 6 | 1 | 45 | PAM, MOXD1 |
|  |  | chemokine activity | 46 | 2 | 46 | IL8, CXCL11, CXCL10 |
|  |  | receptor activity | 1838 | 16 | 49 | HLA-DQB1, PTPRB, OR2A4, OR2A7, PTPRG, OR4F21, IL1RN, KLRK1, OR4F16, NEO1, HLA-DQA2, HLA-DQA1, CD97, CD84, OR4C13, CD86, LILRB4, SCARB1, ELTD1, EMR2, HTR2B, HTR1F |
|  |  | lipid binding | 450 | 6 | 49 | LPL, PLEK, APOD, PLA2G7, VNN1, FABP4, SCARB1, FABP5 |
|  | Up in female | ion binding | 4241 | 39 | 34 | NOX4, ZMAT1, KDM6A, IMPA2, PDP2, NUDT12, ZFX, ZNF354B, CACNG4, COLEC12, ZNF674, ZNF717, ZSCAN16, PHOSPHO1, SLC13A4, CASQ1, KDM5C |

Significance level was set to p <0.05 for both genes and processes. FC = fold-change is the ratio of mean expression for male vs. female cells; score = number of genes after enrichment involved in the respective molecular function and related to genes that show sex-biased expression. The proportion (%) refers to the amount of sex-biased genes found to play a role in the respective molecular function. FDR = false discovery rate.
