# Supplementary material for: The Human Placental Sexome Differs between Trophoblast Epithelium and Villous Vessel Endothelium
Source: PLoS One. 2013 Oct 29;8(10):e79233. doi: 10.1371/journal.pone.0079233 (PMC3812163; doi:10.1371/journal.pone.0079233)
Supplement: Table S8 — Top networks generated using Ingenuity Pathway Analysis with the highest enrichment score of the differentially expressed genes (p<0.05, FC >1.3). (DOCX) [file pone.0079233.s014.docx]

**Table S8. Top networks generated using Ingenuity Pathway Analysis with the highest enrichment score of the differentially expressed genes (p <0.05, FC >1.3).**

|  | **Top Functions** | **Score** | **Focus  molecules** |
| --- | --- | --- | --- |
| **Villous vessel endothelium** | Organismal Injury and Abnormalities, Hereditary Disorder, Respiratory Disease | 27 | 15 |
|  | Cellular Movement, Hematological System Development and Function, Immune Cell Trafficking | 18 | 11 |
|  | Cell Cycle, Cellular Assembly and Organization, Tissue Morphology | 2 | 1 |
| **Trophoblast epithelium** | Cellular Movement, Hair and Skin Development and function, Cancer Cell Death, Tumor Morphology | 30 | 20 |
|  | Cellular Development, Hematological System Development and Function, Hematopoesis | 16 | 13 |
|  | Lipid Metabolism, Small Molecule Biochemistry, Connective Tissue Disorders | 16 | 12 |
|  | Cell Death, Cell-To-Cell Signaling and Interaction, Antigen Presentation | 14 | 12 |
|  | DNA Replication, Recombination and Repair, Cell Death, Cell Cycle | 11 | 10 |

FC = fold-change is the ratio of mean expression for male vs. female cells; score = number of genes after enrichment involved in the respective molecular function and related to genes that show sex-biased expression.
