# Supplementary material for: The Human Placental Sexome Differs between Trophoblast Epithelium and Villous Vessel Endothelium
Source: PLoS One. 2013 Oct 29;8(10):e79233. doi: 10.1371/journal.pone.0079233 (PMC3812163; doi:10.1371/journal.pone.0079233)
Supplement: Table S9 — List of regulators whose downstream target genes are differentially expressed between male and female trophoblast epithelium identified using Ingenuity Pathway Analysis. (DOCX) [file pone.0079233.s015.docx]

**Table S9. List of regulators whose downstream target genes are differentially expressed between male and female trophoblast epithelium identified using Ingenuity Pathway Analysis.**

| **Molecule** | **Molecule type** | **Predicted activation state** | **Regulation z-score** | **Overlap p-value** | **Target molecules** |
| --- | --- | --- | --- | --- | --- |
| IL1RN | cytokine | inhibited | -2.2 | 3.9E-04 | CXCL11, ERAP2, HLA-DQB1, MMP1, TNFSF10 |
| IL1B | cytokine | activated | 2.8 | 9.7E-08 | ACPP, CXCL10, CXCL11, FABP5, IL1RN, IL8, MMP1, MMP3, MMP9, SRGN |
| RELA | transcription regulator | activated | 2.2 | 1.2E-07 | CXCL10, CXCL11, ERAP2, HAS2, IL1RN, IL8, MMP1, MMP9, TGFB1 |
| NFkB (complex) | complex | activated | 2.8 | 6.6E-07 | CD86, CXCL10, ERAP2, HAS2, IL8, MMP1, MMP3, MMP9, TNFSF10 |
| P38 MAPK | group | activated | 2.8 | 2.4E-06 | CD86, CXCL10, IL8, MMP1, MMP3, MMP9, TGFB1, TNFSF10 |
| Jnk | group | activated | 2.4 | 4.8E-06 | CD86, CDH2, GJA1, IL8, MMP1, MMP3 |
| IFNG | cytokine | activated | 2.1 | 7.1E-06 | CD86, CXCL10, CXCL11, ERAP2, HLA-DQB1, IGFBP4, IL1RN, IL8, MMP1, MMP9 |
| CCL5 | cytokine | activated | 2.2 | 3.7E-05 | CD97, CYP1B1, EMP1, IL8, MMP9 |
| IL18 | cytokine | activated | 2.2 | 3.7E-05 | IL8, MMP1, MMP3, MMP9, SMAD3 |
| TNF | cytokine | activated | 3.0 | 9.1E-05 | CD86, CXCL10, CXCL11, IL1RN, IL8, L SS, MMP1, MMP3, MMP9, PRDM1 |
| IL1A | cytokine | activated | 2.2 | 2.8E-04 | CXCL10, IL8, MMP1, MMP10, TGFB1 |
| TREM1 | other | activated | 2.4 | 1.2E-03 | ARRDC4, GREM1, IL8, LPL, MMP1, MMP10, NRIP3 |

IL1RN was the only upstream regulator that shows sex-bias. No upstream regulator has been identified for sex-biased genes in the villous vessel endothelium.
