# Supplementary material for: The Human Placental Sexome Differs between Trophoblast Epithelium and Villous Vessel Endothelium
Source: PLoS One. 2013 Oct 29;8(10):e79233. doi: 10.1371/journal.pone.0079233 (PMC3812163; doi:10.1371/journal.pone.0079233)
Supplement: Table S10 — Metabolic and signaling pathways identified by Pathway studio for placental villi. (DOCX) [file pone.0079233.s016.docx]

**Table S10. Metabolic and signaling pathways identified by Pathway studio for placental villi.**

| **Pathways** | **p-value** | **Genes** |
| --- | --- | --- |
| Insulin Action | 0.001 | GK2, EIF1B, MRPL2, MRPS7, MRTO4, MRPS10, MRPL44, RPS4Y2, RPS6KB1, PRKCQ, GIPC1, SDHD, EIF5A,IGFBP4,IGFBP6,PRKCI, HSD17B10, ACSL3,DLD, PRKAR2A, GSK3A, SLC25A10, ADH1B, FOXD3, MAPK6, PRKX, EEF1B2, APOC4, EIF1AX, EIF1AY, SYT2, CEBPG, UGT2B10, IRS4, HNF4G, ABCD3, TARS, SP6, ACOT8, ACSS1, EPS15L1, RPS4X, SLC27A2, PPP2R2A, MRPS12, RPL9, RSL24D1, SDPR, RPS27, NR2F6, RPS4Y1, NARS, DDX18, RPS21, EIF2S3, AMY1A, YY2, EARS2, MRPL24 |
| B Cell Activation | 0.002 | EIF1B, NLGN4Y, MRPL2, MRPS7, MRTO4, MRPS10, MRPL44, RPS4Y2, CDC42, FN1, PRKCQ, GIPC1, FYN, EIF5A, PRKCI, NFATC1, RAB27A, GP1BA, GGTLC2, CD99, MAPK6, ZAK, EEF1B2, LGALS4, VAMP7, EIF1AX, EIF1AY, SYT2, CEBPG, PPP3R1, TARS, MAP3K10, EPS15L1, RPS4X, PPP2R2A, MRPS12, RPL9, RSL24D1, TAB3, RPS27, CBR3, PCDH11X, RPS4Y1, MPZL2, CHST2, NARS,GGT5, ITPKC, RPS21, CYP2C18, PCDH11Y, EIF2S3,EARS2,MRPL24 |
| Mitochondrial Protein Transport | 0.006 | DNAJA3, FKBP3, PIN4, TBCA, DNAJB2, DNAJA2, PAM16, PPIE, DNAJC8, PMPCA |
| Notch -> MEF/MYOD Signaling | 0.011 | MEF2C, APH1A, APH1B |
| Histone Ubiquitylation | 0.025 | HMGN4,UBE2Z,SIRT3,MECP2,PRM1,TNP2,UBE2M,HMGA2,UBA1,UBE3A, CDC34, HDAC8, ACTL6A, NAE1, MBD1, UBE2J2, CHMP1A, CDY1, POLR2J |
| Cell Cycle Regulation | 0.030 | EIF1B, HMGN4, MRPL2, MRPS7, MRTO4, MRPS10, MRPL44, RPS4Y2, GJB7, GJA1, APP, CDC42, CCNE1, PRKCQ, SIRT3, NPM1, FYN, MECP2, EIF5A, FGF1, E2F4, CELF2, IRAK1, PRKCI, DAXX, TNFRSF10A, PDGFRA, FZR1, CD70, RAP1A, TNFSF13B, PDGFD, PRM1, TNFRSF12A, SURF6, NODAL, TNP2, HMGA2, IPO13, APTX, PRKAR2A, GSK3A, PLEC, MAGOH, ACVR2A, MAPK6, SNRPE, ZAK, PRKX, EEF1B2, HDAC8, PAPOLA, BOP1, EIF1AX, EIF1AY, SLBP, PAFAH1B1, ACTL6A, TUBB4, SMC1A, TES, MBD1, SUPT5H, TBCA, PPIH, TARS, BRF2, CHMP1A, KRT6A, MAP3K10, RPS4X, STAU2, PPP2R2A, TAF9, DEDD2, MRPS12, PPIL1, MAPRE1, KATNA1, RPL9, RSL24D1, TAB3, REXO4, TUBE1, OXR1, MED8, RPS27, MAGEA2, CDY1, ANAPC11, MPHOSPH10, TAF3, POLR2J, TCEA1, RPS4Y1, NARS, MED27, RPS21, EIF2S3, GEMIN8, CENPT, SNRPG, GJB5, EARS2, ZRSR2, MED20, TMED1, NARF, MRPL24, SNRPB2 |
| PDGFR -> STAT Signaling | 0.036 | PDGFRA, PDGFD |
| ActivinR -> SMAD2/3 Signaling | 0.042 | SMURF2, NODAL, ACVR2A |

Genes significantly (p <0.05) differentially expressed between males and females in the placental villi were used for pathway enrichment by Pathway studio. The FC filter of >1.3 was not applied.

FC = fold-change is the ratio of mean expression for male vs. female cells.
